# CFTR Delivery to 25% of Surface Epithelial Cells Restores Normal Rates of Mucus Transport to Human Cystic Fibrosis Airway Epithelium

**DOI:** 10.1371/journal.pbio.1000155

**Published:** 2009-07-21

**Authors:** Liqun Zhang, Brian Button, Sherif E. Gabriel, Susan Burkett, Yu Yan, Mario H. Skiadopoulos, Yan Li Dang, Leatrice N. Vogel, Tristan McKay, April Mengos, Richard C. Boucher, Peter L. Collins, Raymond J. Pickles

**Affiliations:** 1 CF/Pulmonary Research and Treatment Center, University of North Carolina at Chapel Hill, Chapel Hill, North Carolina, United States of America; 2 Respiratory Viruses Section, Laboratory of Infectious Diseases, National Institute of Allergy and Infectious Diseases, National Institutes of Health, Bethesda, Maryland, United States of America; 3 Mayo Clinic College of Medicine, Scottsdale, Arizona, United States of America; 4 Microbiology and Immunology, University of North Carolina at Chapel Hill, Chapel Hill, North Carolina, United States of America; Karolinska Institute, Sweden

## Abstract

Dysfunction of CFTR in cystic fibrosis (CF) airway epithelium perturbs the normal regulation of ion transport, leading to a reduced volume of airway surface liquid (ASL), mucus dehydration, decreased mucus transport, and mucus plugging of the airways. CFTR is normally expressed in ciliated epithelial cells of the surface and submucosal gland ductal epithelium and submucosal gland acinar cells. Critical questions for the development of gene transfer strategies for CF airway disease are what airway regions require CFTR function and how many epithelial cells require CFTR expression to restore normal ASL volume regulation and mucus transport to CF airway epithelium? An in vitro model of human CF ciliated surface airway epithelium (CF HAE) was used to test whether a human parainfluenza virus (PIV) vector engineered to express *CFTR* (PIVCFTR) could deliver sufficient CFTR to CF HAE to restore mucus transport, thus correcting the CF phenotype. PIVCFTR delivered CFTR to >60% of airway surface epithelial cells and expressed CFTR protein in CF HAE approximately 100-fold over endogenous levels in non-CF HAE. This efficiency of CFTR delivery fully corrected the basic bioelectric defects of Cl^−^ and Na^+^ epithelial ion transport and restored ASL volume regulation and mucus transport to levels approaching those of non-CF HAE. To determine the numbers of CF HAE surface epithelial cells required to express CFTR for restoration of mucus transport to normal levels, different amounts of PIVCFTR were used to express CFTR in 3%–65% of the surface epithelial cells of CF HAE and correlated to increasing ASL volumes and mucus transport rates. These data demonstrate for the first time, to our knowledge, that restoration of normal mucus transport rates in CF HAE was achieved after CFTR delivery to 25% of surface epithelial cells. In vivo experimentation in appropriate models will be required to determine what level of mucus transport will afford clinical benefit to CF patients, but we predict that a future goal for corrective gene transfer to the CF human airways in vivo would attempt to target at least 25% of surface epithelial cells to achieve mucus transport rates comparable to those in non-CF airways.

## Abstract 2

Delivering CFTR to ciliated cells of cystic fibrosis (CF) patients fully restores ion and fluid transport to the lumenal surface of airway epithelium and returns mucus transport rates to those of non-CF airways.

## Author Summary

The ciliated epithelium that lines the conducting airways of the lung normally functions to transport hydrated mucus secretions out of the airways to maintain respiratory sterility. Cystic fibrosis (CF) lung disease results from reduced airway surface hydration leading to decreased mucus clearance that precipitates bacterial infection and progressive obstructive lung disease. CF is a genetic disease, and the mutant protein is a chloride ion channel (CFTR) that normally regulates ion and fluid transport on the airway surface. Restoration of corrected CFTR function to the airway epithelium of CF patients by delivering a new *CFTR* gene to airway epithelial cells has long been envisioned as a therapeutic strategy for CF lung disease. Towards this goal, we use a novel viral vector to deliver *CFTR* to a culture model that represents the ciliated airway epithelium of CF patients and show that this strategy restores airway surface hydration and mucus transport to levels of that in non-CF individuals. This study demonstrates efficient and efficacious *CFTR* delivery to CF ciliated airway epithelium and that *CFTR* delivered to approximately 25% of the surface epithelial cells restores normal levels of airway surface hydration and mucus transport. These studies serve as a benchmark for the efficiency of *CFTR* gene delivery to CF airways for future CF gene therapy studies in vivo.

## Introduction

Cystic fibrosis (CF) is the most common recessive lethal genetic disorder in Caucasian populations and results from a defect in the *CFTR* gene. Although CF affects many organs, the pulmonary manifestations account for over 90% of the morbidity and mortality [1]. Dysfunction of CFTR in CF airway epithelium perturbs the normal regulation of ion transport, leading to a reduced volume of airway surface liquid (ASL), mucus dehydration, decreased mucus transport (MCT), and mucus plugging of the airways, which are hallmarks of early CF lung disease. Failure of effective mucus clearance initiates and exacerbates CF lung disease, resulting in an inability to effectively prevent or eradicate bacterial infection, typically dominated by *Pseudomonas aeruginosa*. Persistent neutrophil-mediated inflammation in CF airways further compromises defective clearance and, over several decades, results in airway destruction and fatal decline of lung function.

Airway mucus clearance is dependent on MCT facilitated by ciliated cell function and cough clearance, and constitutes “mechanical” innate defense of the lung [2]. Both MCT and cough clearance require sufficient hydration of mucus secretions for effective airway clearance. The currently accepted model for CF lung pathogenesis is that absence of CFTR function leads to ASL volume reduction that results in mucostasis. A logical therapeutic strategy to reverse CF lung pathogenesis would therefore replace CFTR function to CF airway epithelium, restoring ASL volume regulation and MCT. *CFTR* gene delivery strategies remain a rational approach towards this goal. To date, however, clinical trials in CF patients using *CFTR* gene delivery techniques have resulted in limited successful gene delivery that is widely considered to be insufficient for therapeutic benefit to CF patients. The fundamental hurdle to these approaches is the low efficiency of *CFTR* gene delivery to human conducting airway epithelial cells that regulate MCT.

In non-CF human airways, CFTR is expressed in ciliated airway epithelial cells of the surface and submucosal gland ductal epithelium [3], and in the fluid-secreting cells of the submucosal glands [4]. Ciliated cells are the predominant luminal epithelial cell type present throughout the proximal and distal conducting airways and are critical cell types for facilitating MCT [5]. These properties of ciliated cells make them an abundant and relevant target for *CFTR* gene delivery. Since restoration of MCT or ASL volumes to normal non-CF levels has not been described after delivery of CFTR to CF airway epithelium either in vitro or in vivo, it remains unknown how much CFTR or how many surface epithelial cells will be required to express CFTR to restore mechanical innate defense to the airways.

In vitro models of human ciliated airway epithelium (HAE) recapitulate the morphology and physiology of the human airway epithelium and have been a valuable tool in the study of cell physiologic mechanisms that regulate ion and fluid transport and MCT in the human ciliated conducting airways [6]–[8]. Importantly, HAE retain phenotypic differences between non-CF and CF airway epithelium, i.e., CF HAE exhibit reduced or absent cAMP-mediated chloride ion (Cl^−^) secretion, dysregulation of sodium ion (Na^+^) absorption, excessive ASL absorption, dehydration of secreted mucus, and mucostasis [6]. This model has been predictive of the in vivo efficacy of drug- and gene-based therapeutics in human clinical studies [9]–[16]. In particular, this model has been utilized to determine that currently available gene transfer vectors approved for clinical testing (e.g., adenovirus, lentivirus, AAV, and nonviral vectors) are inefficient at delivering CFTR to sufficient numbers of surface epithelial cells to restore CFTR function or ASL volume for effective MCT. The target number of cells in a ciliated airway epithelium needed to express CFTR for restoration of ASL and MCT is currently unknown.

We have shown that human parainfluenza virus (PIV) selectively targets ciliated cells of HAE after luminal delivery [17]. Since recombinant PIV can be re-engineered to express large transgene inserts [18], we used PIV to: (1) generate PIV-expressing *CFTR* as an additional gene (PIVCFTR); (2) test whether delivery of *CFTR* to CF ciliated cells restored mechanical innate defense, i.e., MCT, to human CF airway epithelia; and (3) determine the numbers of CF epithelial cells requiring CFTR to restore MCT rates to normal non-CF HAE levels. Using CF HAE, CFTR expression levels per cell and numbers of cells expressing CFTR were correlated with correction of ion transport, ASL volume regulation, and MCT rates to assess the relationship between gene transduction and restoration of normal mucociliary transport. We show that PIV-mediated delivery of CFTR to ciliated cells of CF HAE resulted in functional CFTR channel activity with restoration of ASL volume homeostasis and MCT. Further, we show that CFTR expression in individual ciliated cells does not require tight regulation of expression and that restoration of MCT rates to those measured in non-CF HAE required CFTR delivery to at least 25% of surface epithelial cells or approximately 30% of ciliated cells. Hence, we describe the first demonstration, to our knowledge, of efficient *CFTR* gene delivery to CF ciliated airway epithelium that is sufficient to correct the fundamental physiological dysfunctions that precipitate CF lung pathogenesis.

## Results

### Efficient CFTR Delivery to Ciliated Airway Epithelium by PIV

**Figure 1:**
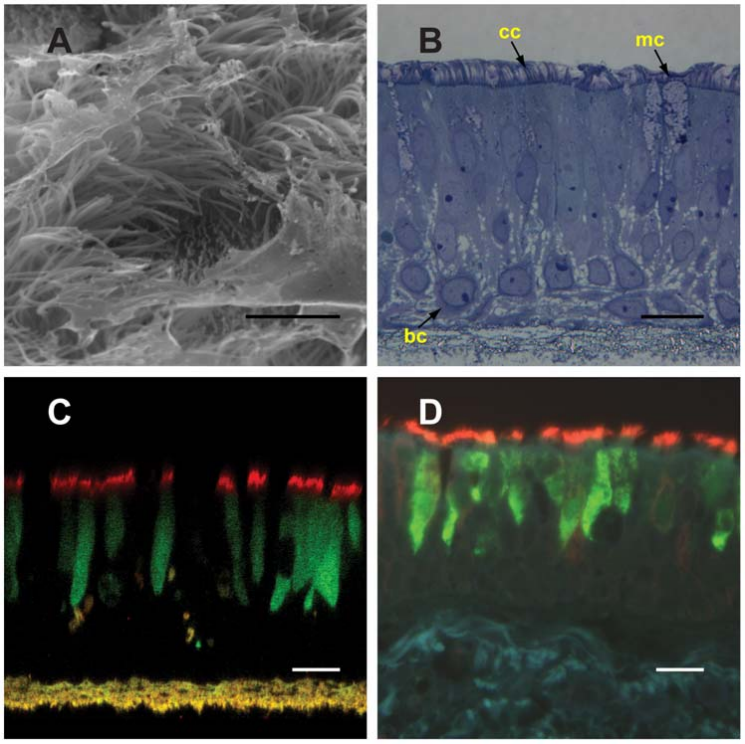
Infection of human ciliated cells by PIV in vitro and ex vivo. (A) Scanning electron micrograph of HAE showing cilia and mucus on the luminal surface. (B) Histological cross-section of HAE showing pseudostratified, columnar airway epithelium with ciliated (cc) and mucin-secreting cells (mc) and basal epithelial cells (bc). (C) Representative confocal *XZ* sections of HAE or (D) histological sections of human tracheobronchial tissue inoculated with PIVGFP (10^6^ PFU) and GFP expression assessed at 24 h pi. GFP was detected by indirect immunofluorescence with rabbit anti-GFP polyclonal antibodies (Ab) and goat anti-rabbit IgG-fluorescein (green). Ciliated cells were identified using mouse primary Ab against acetylated α-tubulin and detected with anti-mouse IgG-Texas Red (red). GFP colocalized to cells that were also positive for acetylated α-tubulin, confirming the targeting of ciliated cells in vitro and ex vivo by PIV. Bar represents 20 µm in (A); and 5 µm in (B, C, and D).

Recombinant PIV-expressing GFP (PIVGFP) infects ciliated cells of an in vitro model of human airway epithelium that recapitulates the morphology of the human ciliated airway epithelium in vivo (Figure 1A, 1B, and 1C). Inoculation of freshly excised human tracheobronchial airway epithelium showed PIVGFP also targeted ciliated cells under noncultured conditions (Figure 1D).

**Figure 2:**
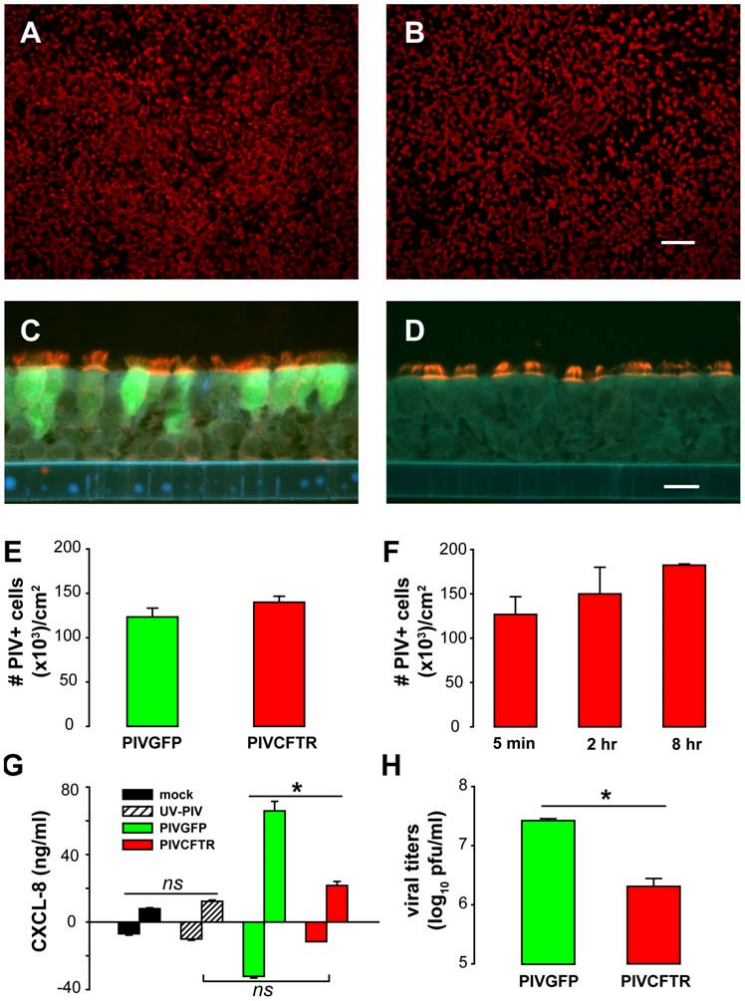
Efficient targeting of CF ciliated cells by PIVCFTR. (A–D) Representative immunodetection of human PIV fusion (F) glycoprotein (red) (A and B) en face or (C and D) in histological sections of CF HAE 48 h pi with PIVGFP (A and C) or PIVCFTR (B and D). F glycoprotein was detected by indirect immunofluorescence with a murine monoclonal anti-F Ab and anti-mouse IgG conjugated to AlexaFluor 594 (red). GFP-positive cells were identified in histological sections (C), using rabbit anti-GFP conjugated to fluorescein (green). Fluorescence was viewed using a Texas Red filter only (A and B) or a combined Texas Red-FITC-UV-filter (C and D). Bars represent 200 µm and 10 µm for (A and B, and C and D), respectively. (E and F) Quantitation of (E) numbers of PIV F-positive ciliated cells showing that PIVGFP and PIVCFTR infect similar numbers of ciliated cells at 48 h pi (*n* = 8); and (F) the efficiency of ciliated cell infection by PIVCFTR assessed at 24 h was similar with 5-min, 2-h, or 8-h inoculation times (*n* = 6). (G) CXCL8 protein levels secreted by HAE 48 h pi with mock, UV-inactivated PIV, PIVGFP, or PIVCFTR showing reduced levels for PIVCFTR versus PIVGFP. Responses positive/negative of the abscissa reflect CXCL8 secretion into apical/basolateral compartments, respectively (*n* = 5 for each). (H) Viral titers in apical compartment at 48 h pi for PIVGFP (green) and PIVCFTR (red) showing attenuated growth of PIVCFTR (*n* = 5 for each). An asterisk (*) denotes *p*<0.05; ns, not significantly different. Error bars indicate SEM .

To express CFTR in CF ciliated cells, a PIV with *CFTR* inserted into the viral genome was constructed (PIVCFTR, Figure S1). Apical surface inoculation of CF HAE with PIVCFTR or PIVGFP at 10^6^ plaque-forming units (PFU) (100 µl of 10^7^ PFU/ml for 2 h: multiplicity of infection [MOI] ∼3 for all lumenal cells and ∼5 for ciliated cells) resulted in infection of a significant number of cells 48 h postinoculation (pi) as detected by immunolocalization of PIV fusion (F) glycoprotein viewed en face (Figure 2A and 2B). Immunolocalization of PIV-mediated GFP and PIV F expressed by PIVGFP or F glycoprotein expressed by PIVCFTR confirmed targeting of ciliated cells (Figure 2C and 2D). Quantitation of lumenal cells infected by PIVGFP or PIVCFTR revealed that similar numbers of ciliated cells were infected by each virus (Figure 2E). Although ciliated cell numbers were variable in HAE derived from different donors (range 60% to 80%), the percentage of ciliated cells per square centimeter of epithelium surface area determined by β-tubulin IV immunodetection showed that on average approximately 70% of surface cells were ciliated cells. Since the total number of lumenal surface cells in HAE approximates 0.3×10^6^ cells, we estimate that approximately 42% of surface cells or approximately 60% of ciliated cells were infected by PIV under these conditions.

The PIV vector replicates in ciliated cells and by 24 h pi, sheds progeny virions onto the culture surface, resulting in further rounds of ciliated cell infection. However, when low viral titers (10^3^ PFU) were used to inoculate HAE, evidence of further rounds of infection (i.e., spread) was not apparent until 48 h pi [17]. To determine whether the high efficiency of ciliated cell infection by PIVCFTR was dependent on spread of progeny virus from ciliated cell to ciliated cell, we compared infection rates at 24 h versus 48 h pi. HAE inoculated with PIVCFTR (10^6^ PFU) for 2 h showed similar high infection rates at 24 h pi as for 48 h (2-h data for Figure 2F vs. 2E), indicating that the initial high titer inoculum and not cell–cell spread of virus mediated the highly efficient targeting of ciliated cells by PIV. Prolonging or decreasing inoculation times to 8 h or 5 min produced modestly increased or decreased infection rates, respectively (Figure 2F). The high numbers of PIVCFTR-infected cells with only 5-min inoculation time highlight the high efficiency of PIV infection and suggest that short exposure times in the airways will be sufficient for efficient targeting of ciliated cells in vivo.

Although equally efficient at targeting ciliated cells as PIVGFP, PIVCFTR stimulated significantly lower amounts of epithelial cell-derived inflammatory mediators associated with in vivo viral pathogenesis. Indeed, PIVCFTR only induced inflammatory mediator secretion to levels stimulated by inoculation with UV-inactivated PIV (Figure 2G for CXCL8, and Figure S2 for CXCL10, IL-6, IL-12p40, MCP-1, and RANTES). Since PIVCFTR produced 10-fold fewer progeny virions than PIVGFP due to the insertion of the relatively large *CFTR* insert (Figure 2H), it is likely that the generation of inflammatory mediators is proportional to the rate of PIV replication in ciliated cells.

### PIV Delivers CFTR to Ciliated Cells, Resulting in Overexpression and Apical Localization of Functional CFTR

**Figure 3:**
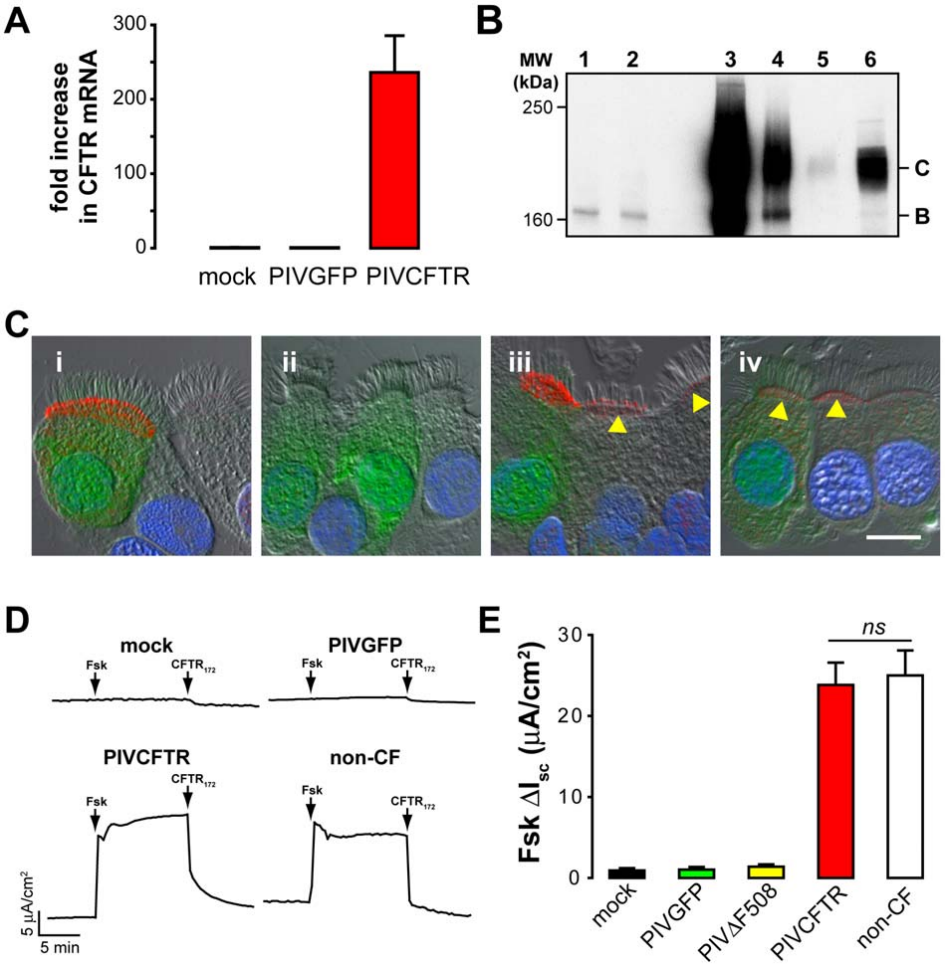
Expression of functional CFTR in CF ciliated cells targeted by PIV. (A) *CFTR* mRNA levels in CF HAE 48 h after inoculation with PIVGFP or PIVCFTR and relative to mRNA levels in CF HAE mock-inoculated (*n* = 12). (B) Representative western blot of CFTR protein in lysates of CF HAE 48 h pi with mock (lane 1), PIVGFP (lane 2), and PIVCFTR (lanes 3–5), with lanes 4 and 5 representing serial 10-fold dilution of cell lysates. For comparison, non-CF HAE lysates also were included to detect endogenous CFTR levels (lane 6). Markers indicate the fully glycosylated mature form of CFTR (Band C) and immature nonglycosylated CFTR (Band B). Data are representative of experiments with cells derived from two separate patients. (C) Representative confocal images of CFTR immunoreactivity in CF HAE (i and ii), and non-CF HAE (iii and iv), 48 h after inoculation with PIVGFPCFTR (i and iii) or PIVGFP (ii and iv). CFTR was detected with CFTR monoclonal Ab (clone 596) and secondary Abs conjugated to AlexaFluor 594 (red). CF HAE inoculated with PIVGFPCFTR (i) showed immunolocalization of CFTR at apical domains of ciliated cells that were also GFP-positive, but not in GFP-negative ciliated cells or in CF HAE infected with PIVGFP (ii). Robust apical domain CFTR and GFP were detected in non-CF HAE ciliated cells inoculated with PIVGFPCFTR and endogenous CFTR levels detected in GFP-negative ciliated cells (iii, arrowheads). Endogenous CFTR was detected at the apical membranes of GFP-positive and -negative ciliated cells after inoculation with PIVGFP (iv, arrowheads). Bar represents 5 µm. (D) Representative traces of short-circuit current measurements (*I*_sc_) from CF HAE 48 h pi by PIVCFTR, PIVGFP, or mock showing post amiloride responses to sequentially added forskolin (Fsk), and CFTR_172_. A representative Fsk-induced *I*_sc_ response by a non-CF HAE is shown for comparison. No Fsk responses were seen in CF HAE inoculated with PIVGFP or mock, consistent with the absence of CFTR. (E) Summary data for Fsk-activated changes in *I*_sc_ (Δ*I*_sc_) in CF HAE 48 h pi with mock, PIVGFP, PIVΔF508CFTR, or PIVCFTR. Fsk responses for non-CF HAE are shown as comparison. CFTR delivery to ciliated cells resulted in restoration of functional Cl^−^ channel activity in CF HAE to levels exhibited in non-CF HAE. Each bar represents at least 11 cultures from four different patients. ns denotes not significantly different. Error bars indicate SEM.

Expression levels of transduced CFTR in CF HAE were determined by comparing the levels of exogenous *CFTR* mRNA expressed by PIVCFTR relative to endogenous *CFTR* mRNA in CF HAE and non-CF HAE using quantitative RT-PCR. Previously, it has been estimated that human airway cells contain only approximately 10 *CFTR* transcripts/cell [19]. In our experiments, we found that CF HAE inoculated with PIVCFTR produced a 236-fold increase in *CFTR* mRNA when compared to cultures inoculated with PIVGFP or mock (Figure 3A). Since this large-fold increase in *CFTR* mRNA is in part reflective of the low endogenous copy number of *CFTR*, we also assessed CFTR protein levels semiquantitatively by western blot. CF HAE inoculated with PIVCFTR expressed large amounts of mature CFTR (Figure 3B, lane 3, band C), whereas no mature CFTR protein was detected in CF HAE inoculated with vehicle alone (lane 1) or PIVGFP (lane 2). Serial 10-fold dilutions of total protein lysates of CF HAE inoculated with PIVCFTR (lanes 4 and 5) provided a semiquantitative measurement of the amounts of exogenous CFTR protein in CF HAE compared to CFTR protein levels in non-CF HAE (lane 6). We estimate that an approximately 50-fold increase in mature CFTR protein in transduced CF HAE was achieved compared to non-CF HAE. Therefore, two independent measures of CFTR abundance indicate a significant overexpression of both *CFTR* mRNA and protein in transduced CF HAE. Note, these measures are likely an underestimate given that not all ciliated cells are infected by PIVCFTR. Because approximately 60% of ciliated cells were infected in these experiments, and ciliated cells on average comprise approximately 70% of surface cells within a culture, we estimate that individual infected ciliated cells likely overexpress CFTR protein by at least 100-fold over non-CF ciliated cells.

Apical localization and overexpression of CFTR above endogenous levels in ciliated cells was confirmed by immunodetection of CFTR in CF HAE and non-CF HAE (Figure 3C). For these studies, we chose to engineer both *GFP* and *CFTR* into a single PIV vector (PIVGFPCFTR) to enable identification of infected cells by GFP fluorescence. In CF HAE infected with PIVGFPCFTR, CFTR was immunolocalized only to ciliated cells that were also positive for GFP (Figure 3Ci) and concentrated in apical membrane domains at the base of the cilial shafts. Although endogenous CFTR in non-CF ciliated cells in vitro is localized to these regions [3], subapical membrane CFTR immunoreactivity was also detected after PIVGFPCFTR, likely suggesting the increased presence of CFTR in recycling endosomes. Infection of CF HAE with PIVGFP alone showed that ciliated cells positive for GFP were negative for CFTR immunoreactivity (Figure 3Cii). When non-CF HAE were infected by PIVGFPCFTR, endogenous CFTR was present in GFP-negative ciliated cells and overexpressed in GFP-positive cells (Figure 3Ciii). For non-CF HAE infected with PIVGFP, GFP-positive and -negative ciliated cells showed only endogenous CFTR apical membrane immunoreactivity (Figure 3Civ). CFTR (endogenous or PIV-delivered), GFP, or PIV antigens were never detected in cell types that did not posses cilia (Figure S3).

To determine whether PIV-mediated CFTR delivery to ciliated cells resulted in functional CFTR anion channel activity in CF HAE, we maximally stimulated cAMP-mediated anion transport capacity using forskolin (Fsk), an activator of CFTR. Figure 3D shows bioelectric short-circuit current (*I*_sc_) traces obtained in Ussing chamber experiments with CF HAE inoculated with mock (vehicle alone), PIVGFP, or PIVCFTR. For comparison, a *I*_sc_ trace from a non-CF HAE is also shown. Whereas *I*_sc_ responses to Fsk were not observed in mock- or PIVGFP-inoculated CF HAE, CF HAE inoculated with PIVCFTR exhibited rapid and sustained increases in *I*_sc_ that were rapidly inhibited by a CFTR-specific inhibitor (CFTR_172_ [20]). Experiments using CF cells derived from four different donors revealed that the kinetics and magnitudes of the Fsk responses in PIVCFTR-corrected CF HAE were indistinguishable from those observed for non-CF HAE (Figure 3D and 3E; range 6.7–42.0 µA/cm^2^ for PIVCFTR, 0–1.5 µA/cm^2^ for CF HAE controls [mock and PIVGFP] and 7.8–70.3 µA/cm^2^ for non-CF HAE). An additional control using PIV expressing the nonfunctional *CFTR* mutant *ΔF508CFTR* (PIVΔF508) confirmed that functional CFTR was required for bioelectric correction of CF HAE (Figure 3E). These data show that delivery of functional CFTR to CF ciliated cells fully restored maximally stimulated CFTR anion channel activity to normal non-CF levels. PIVCFTR did not significantly affect UTP-mediated Cl^−^ secretion in CF HAE beyond that of PIVGFP (Figure S4).

### Overexpression of CFTR in CF Ciliated Cells Does Not Increase Anion Transport Beyond That of Non-CF HAE

Although others have shown that overexpression of CFTR was not detrimental to airway epithelial cell integrity in vitro and in vivo [21],[22], we had anticipated that Fsk-stimulated Cl^−^ secretion would exceed that of non-CF HAE since CF ciliated cells significantly overexpressed CFTR, i.e., CF HAE would be “supercorrected.” That CF HAE overexpressing CFTR exhibited identical anion secretion as measured in non-CF HAE with endogenous CFTR levels suggested that the ceiling for anion secretion rates was not solely related to the absolute quantity of CFTR present in ciliated cells. Several explanations appeared plausible to account for this observation.

A first explanation is that transduced CFTR was not trafficked to the apical membrane of CF ciliated cells. As shown in Figure 3C, this was not the case, as immunofluorescent localization revealed clear targeting of transduced CFTR to apical domains of CF ciliated cells. It is possible, however, that not all correctly trafficked CFTR was inserted into the apical membrane in regions that facilitate function. Certainly, our immunolocalization data suggest CFTR is present in subapical membrane structures likely representing recycling endosomes.

**Figure 4:**
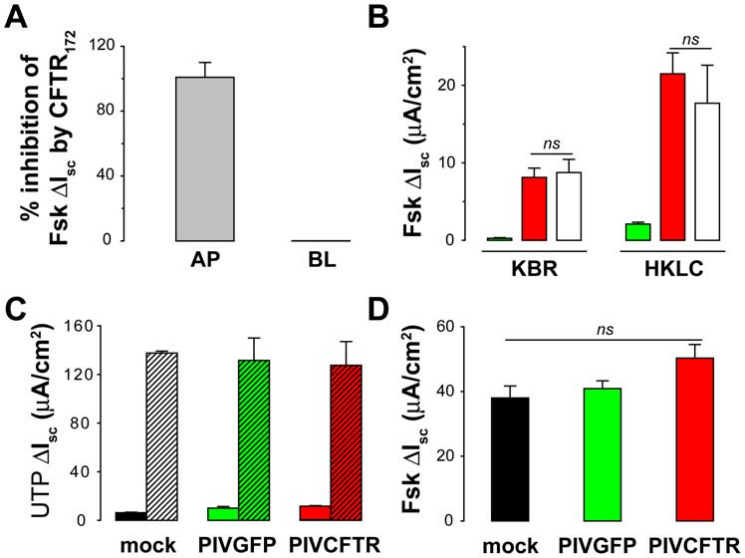
CFTR activity in ciliated cells is regulated by factors other than CFTR levels. (A) Percentage inhibition of Fsk-activated CFTR by CFTR_172_ applied to either the apical (AP) or basolateral (BL) surfaces of PIVCFTR-corrected CF HAE. CFTR activity was inhibited by apical, but not basolateral, addition of CFTR_172_ for at least 15 min (*n* = 4). (B) Driving force for Cl^−^ secretion does not dictate magnitude of the Fsk response determined by measurement of Fsk-mediated Δ*I*_sc_ under physiological Cl^−^ concentration (KBR) or Cl^−^-free solutions (HKLC) in CF HAE 48 h after inoculation by PIVGFP (green bars) or PIVCFTR (red bars) and compared to non-CF HAE (white bars) (*n* = 8). (C) UTP-mediated Cl^−^ secretion in KBR (solid bars) and HKLC (hatched bars) bathing solutions after inoculation of CF HAE with vehicle alone (black bars), PIVGFP (green bars), or PIVCFTR (red bars) (*n* = 6). Note that under conditions of increased driving force for Cl^−^ secretion, responses far exceed the maximal responses achieved with Fsk-mediated CFTR activation in the presence of overexpressed CFTR. (D) Fsk-induced Δ*I*_SC_ in non-CF HAE inoculated with mock (black bars), PIVGFP (green bars), or PIVCFTR (red bars), showing that overexpression of CFTR on top of endogenous CFTR does not significantly enhance the Fsk-mediated secretory response under physiological conditions (*n* = 5 for each). ns denotes not significantly different. Error bars indicate SEM .

A second explanation is that overexpression of CFTR resulted in mislocalization of a fraction of CFTR to basolateral membranes of ciliated cells. This event would be predicted to dampen Fsk-induced CFTR responses as suggested for adenovirus-mediated CFTR delivery to airway epithelia [23]. Although CFTR immunoreactivity was restricted to the apical domains of ciliated cells, even when overexpressed (Figure 3C), we further tested for this possibility by using CFTR_172_ as a probe to measure CFTR functional activity in apical and/or basolateral compartments of PIVCFTR-corrected CF HAE. Addition of CFTR_172_ to apical surfaces 15 min before or during Fsk-induced anion secretion resulted in rapid and complete inhibition of secretion (Figure 4A). In contrast, CFTR_172_ applied to basolateral surfaces before or during Fsk-induced anion secretion had no effect on Fsk-induced anion secretion, suggesting that no significant functional CFTR was present in the basolateral membranes of ciliated cells.

A third explanation is that the Fsk responses are limited by the apical membrane driving force for Cl^−^ secretion. We tested this possibility by comparing the Fsk responses with PIVCFTR-corrected CF HAE (>100-fold increased CFTR) to non-CF HAE (endogenous CFTR levels) with protocols designed to make the electrochemical driving force for Cl^−^ secretion nonlimiting (bathing solutions changed from Cl^−^ replete [Krebs bicarbonate Ringer, KBR] to Cl^−^ deplete [high potassium low chloride, HKLC]). As shown in Figure 4B, Fsk responses in CF HAE overexpressing CFTR and non-CF HAE were similar under conditions of physiological Cl^−^ secretory driving forces (KBR). Importantly, when the apical membrane Cl^−^ secretory driving force was made large and not limiting by lumenal Cl^−^ substitution, Fsk again stimulated similar responses in CF HAE overexpressing CFTR compared to non-CF HAE (Figure 4B, HKLC). These observations strongly suggest that overexpressed levels of CFTR do not produce reduction in Cl^−^ secretory driving forces that offset the additional quantity of CFTR in the apical membrane and hence buffer the Cl^−^ secretion rates. As an additional control, UTP-mediated Cl^−^ secretion (via calcium-activated Cl^−^ channels) was increased >20-fold in HKLC bathing solution compared to KBR (Figure 4C), indicating that the maximal anion secretory capacity of cultures had not been reached.

Collectively, these data suggest that CFTR overexpressed in CF ciliated cells is selectively trafficked to the apical domains of these cells. However, a ceiling of CFTR Cl^−^ secretion is reached that approximates that of endogenous CFTR in non-CF HAE. We speculate that this ceiling reflects the limiting requirement of the number of potential apical membrane docking sites for CFTR and/or the limited amount of accessory/regulatory proteins localized at apical membranes of CF cells required for CFTR function. It is likely that CFTR insertion into the apical membranes of ciliated cells is tightly regulated with recycling and replenishment of CFTR governed by CFTR-rich recycling endosomes. To determine whether limited availability of docking sites or accessory proteins was unique to CF ciliated cells, non-CF HAE were inoculated with PIVCFTR and PIVGFP, and Fsk responses compared (Figure 4D). Overexpression of CFTR in non-CF HAE provided only moderately increased Fsk-mediated Cl^−^ secretion compared to cultures inoculated with PIVGFP. Although modest but significant differences in resistance were measured before/after Fsk treatment in mock-treated non-CF HAE, no significant differences in resistance were measured before/after forskolin treatment after PIVCFTR versus PIVGFP inoculations: (Resistances [Ω.cm^2^] before/after Fsk: Mock, 550±49/401±0.5; PIVGFP, 452±39/402±0; PIVCFTR, 419±12/401±0.2; *n* = 5 for each). Collectively, these data suggest replacement of a corrective *CFTR* gene to CF ciliated cells is the only manipulation required for correction of the CF defect since both CF and non-CF HAE regulate CFTR activity similarly.

### Restoration of ASL Volume Regulation in CF HAE by Expressing CFTR in Ciliated Cells

**Figure 5:**
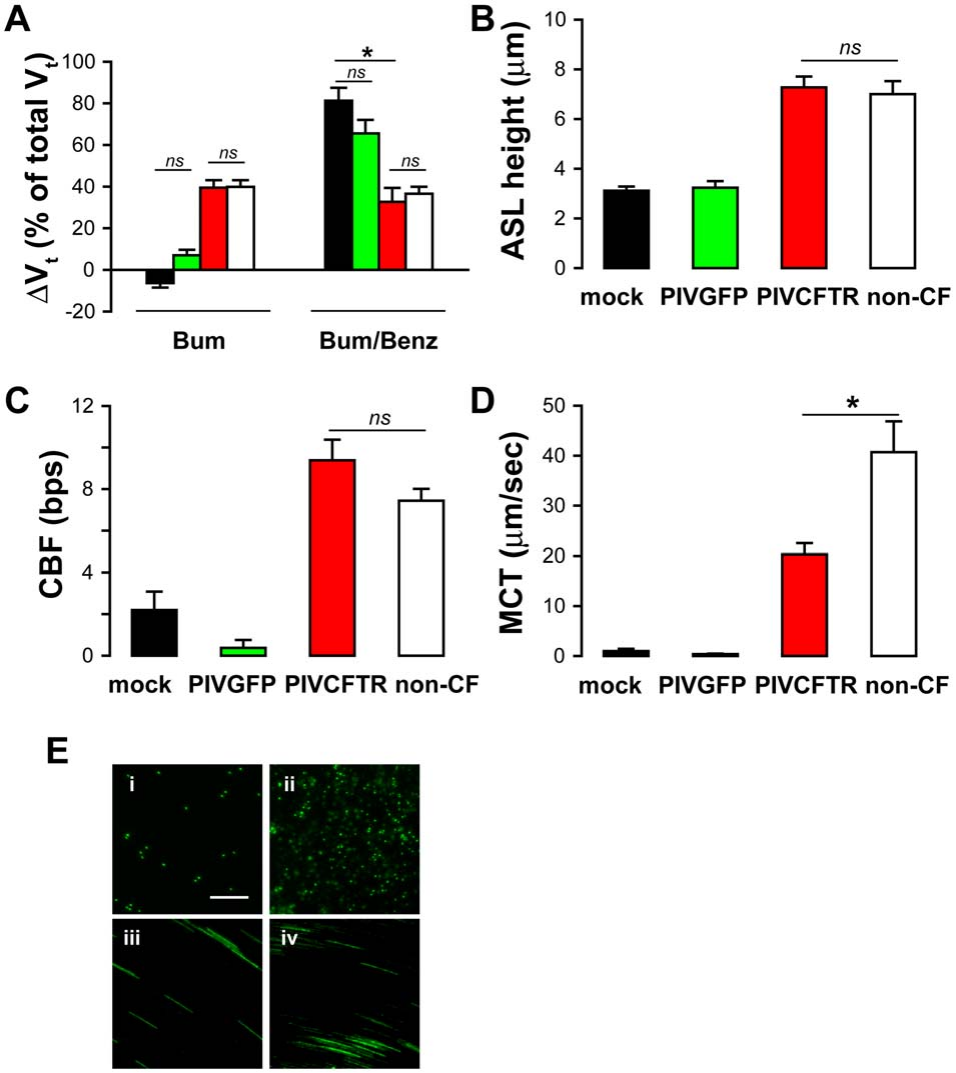
Expression of CFTR in CF ciliated cells restores normal ASL homeostasis and MCT to CF HAE. (A) The contribution of Cl^−^ and Na^+^ to transepithelial electrical potential difference (*V_t_*) under thin-film conditions in CF HAE mock-inoculated (black bars), PIVGFP (green bars), or PIVCFTR (red bars). *V_t_* changes for non-CF HAE are shown for comparison (white bars). Bar graphs depict percentage change in *V_t_* in response to Cl^−^ channel inhibitor bumetanide (Bum) and Na^+^ channel inhibitor benzamil (Bum/Benz). Each bar represents eight cultures derived from three different patients, and an asterisk (*) indicates *p*<0.05, and ns indicates not significant. (B) ASL height measurements 24 h after apical addition of 25 µl of PBS containing Texas Red dextran to CF HAE 48 h pi with mock (black), PIVGFP (green), or PIVCFTR (red) and compared to ASL height in non-CF HAE (white). Each bar represents nine cultures from three patients. (C) Cilia beat frequency measurements (CBF in beats per second [bps]) from CF HAE 24 h after the addition of 25 µl of PBS to CF HAE 48 h pi with mock (black), PIVGFP (green), or PIVCFTR (red) and compared to non-CF HAE (white). Each bar represents six cultures derived from two patients. (D) MCT rates measured 24 h after bead addition to CF HAE inoculated with mock (black), PIVGFP (green), or PIVCFTR (red). MCT for non-CF HAE are shown for comparison (white). Each bar represents at least nine cultures derived from three patients. (E) Representative photomicrographs showing time-lapse (3-s exposures) movement 24 h after addition of green fluorescent microspheres (as an index of MCT) on CF HAE 48 h after inoculation with mock (i), PIVGFP (ii), or PIVCFTR (iii). Note: GFP-positive cells are also observed below beads in PIVGFP-inoculated CF HAE. Bar represents 60 µm, and asterisk (*) denotes *p*<0.05, and ns not significantly different compared to non-CF HAE (iv). Error bars indicate SEM.

The dehydrated airway surface phenotype characteristic of CF results from the inability to induce Cl^−^ secretion and the failure to regulate Na^+^ absorption to maintain ASL height at approximately 7–10 µm, i.e., physiologic “thin-film” volumes. We investigated whether expression of CFTR in CF ciliated cells corrected both the Cl^−^ secretory and Na^+^ hyperabsorptive phenotype of CF HAE by measuring responses to specific antagonists on transepithelial potential difference (*V_t_*) with microelectrodes under thin-film conditions when the height of the ASL was at steady state. Measurement of the basal contribution of Cl^−^ transport to *V_t_* by blocking basolateral membrane cellular Cl^−^ entry with bumetanide (10^−4^ M) showed that 40% of the *V_t_* in non-CF HAE was accounted for by Cl^−^ ion transport (Figure 5A, white bars). In contrast, in CF HAE, there was no detectable bumetanide-sensitive *V_t_*, consistent with the absence of functional CFTR (black bars). However, after PIVCFTR, but not PIVGFP, the contribution of Cl^−^ transport to CF HAE *V_t_* was indistinguishable from that for non-CF HAE (approximately 40%, red bars). These data obtained under thin-film conditions confirm that delivery of CFTR to CF ciliated cells fully corrected the Cl^−^ secretory defect to non-CF levels.

The epithelial Na^+^ channel (ENaC) is rate-limiting for Na^+^ absorption and is negatively regulated by CFTR expression [24]–[26]. Since ENaC activity is regulated by mediators present in the ASL (e.g., nucleotides and proteases [27],[28]), and Ussing chamber (“thick-film”) studies result in washing away of these critical ENaC regulatory factors, we determined how CFTR delivery to CF ciliated cells affected the regulated activity of ENaC under thin-film conditions using microelectrodes. The change in *V_t_*, in response to the Na^+^ channel blocker, benzamil (10^−5^ M), indicated that Na^+^ transport accounted for approximately 40% of the total *V_t_* in non-CF HAE (Figure 5A, white bars) and approximately 80% of transport in CF HAE (black bars), consistent with a Na^+^ hyperabsorptive phenotype for CF airway epithelia. Expression of CFTR, but not GFP, in CF ciliated cells significantly reduced the contribution of CF HAE Na^+^ transport to levels measured in non-CF HAE (Figure 5A, red bars). These data show that delivery of CFTR to CF ciliated cells restored both Cl^−^ secretion and the regulation of Na^+^ absorption by CF airway epithelia to normal, non-CF HAE levels. The simplest conclusion drawn from these data is that ENaC and CFTR both reside in ciliated cells in the human airway epithelium.

### CFTR Expression in CF Ciliated Cells Restores Regulation of Apical Surface Hydration

During these experiments, it was noted that the lumenal surfaces of PIVCFTR-corrected CF HAE appeared hydrated compared to the dehydrated surfaces of CF HAE, suggesting that the rebalancing of Na^+^ absorption and Cl^−^ secretion consequent to delivery of CFTR to ciliated cells restored hydration to the lumenal surfaces of CF HAE. Therefore, we initiated experiments to measure ASL height regulation in CF HAE in the absence or presence of transduced CFTR in ciliated CF cells.

ASL volume regulation was assessed by measuring ASL height with *XZ*-plane confocal microscopy 48 h after addition of 25 µl of PBS to the apical surfaces of CF HAE. In control CF HAE (inoculated with mock or PIVGFP), CF epithelia absorbed almost all fluid from their surfaces, resulting in an ASL height of 3 µm, i.e., the minimal space of compacted folded-over cilia and consistent with mucostasis (Figure 5B) [29]. However, in PIVCFTR-corrected CF HAE, ASL height stabilized at approximately 8 µm, a height similar to that of non-CF HAE (Figure 5B). These data show that delivery of CFTR to CF ciliated cells fully restored the regulation of ASL height to levels maintained by non-CF HAE, thus establishing the critical role of CFTR and ciliated cells in ASL height homeostasis.

We next investigated whether the depletion of ASL over time impaired cilia beat by measuring ciliary beat frequency (CBF) of CF HAE under thin-film conditions. Immediately after addition of 25 µl of PBS to the apical surface of CF HAE, CBF was approximately 8 Hz, a value not different than exhibited by non-CF HAE (Figure 5C). However, consistent with the decreased ASL height in control CF HAE, effective CBF was reduced 48 h later in CF HAE compared to non-CF HAE (Figure 5C). In contrast, effective CBF was maintained in PIVCFTR-inoculated, but not PIVGFP-inoculated, CF HAE at levels similar to those measured in non-CF HAE (Figure 5C). These data strongly argue that ineffective cilia beat observed in CF HAE reflects a defect in ASL height regulation, not ciliary function, and restoration of ASL height regulation with PIVCFTR is sufficient to restore effective cilia beat in CF HAE.

### CFTR Expression in CF Ciliated Cells Restores Effective MCT

A novel feature of the HAE model is the recapitulation of MCT, reflecting coordinated cilia beat that produces rotational flow of mucus over hydrated epithelial apical surfaces [6]. In CF HAE, the rotational flow is abolished due to ASL depletion, mimicking mucostasis described for CF airways in vivo [6]. To determine whether expression of CFTR in CF ciliated cells could prevent mucostasis, CF HAE were inoculated with PIVCFTR, PIVGFP, or mock and, 24 h later, a small bolus of 1-µm fluorescent beads added to the apical surfaces and cultures maintained at >95% humidity for 24 h. For control CF HAE, rotational flow of beads was rarely observed at 48 h pi (Figure 5D, 5Ei, and 5Eii), i.e., mucostasis occurred. In contrast, PIVCFTR-corrected CF HAE exhibited significant rotational flow of beads, indicating that MCT had been restored (Figure 5D and 5Eiii). Under these conditions at 48 h pi, PIVCFTR restored approximately 50% of the MCT measured in parallel non-CF HAE (Figure 5D and 5Eiv). These data are the first demonstration, to our knowledge, that MCT can be restored to CF airway epithelia by delivering CFTR to ciliated cells, indicating that the cumulative effects of CFTR deficiency on mechanical innate defense can be reversed by this strategy.

Why MCT was not completely restored to normal levels, especially when ion transport processes and ASL volume regulation were fully corrected, remains to be determined. A possible explanation may relate to potentially subtle cytopathic effects of PIV infection at 48 h pi. To address this possibility, we inoculated non-CF HAE with PIVGFP or PIVCFTR, and assessed MCT 48 h later. PIVGFP decreased MCT to 40±3% (*n* = 5) of normal levels, whereas PIVCFTR decreased MCT to approximately 74±11% (*n* = 5) of normal levels. These data strongly suggest that after 48 h of PIV infection, virus-induced cytopathic events, likely linked to virus replication capacity, limit complete restoration of MCT to non-CF levels.

**Figure 6:**
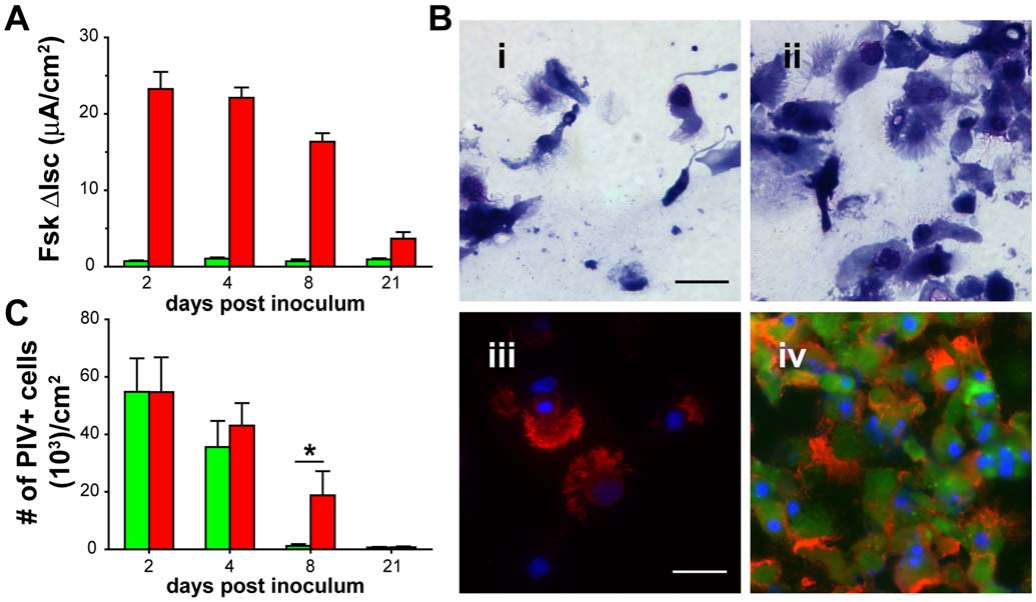
Duration of CFTR functional correction is limited by shedding of PIV-infected ciliated cells. (A) Duration of CFTR functional activity in PIV corrected CF HAE. CF HAE were inoculated with PIVCFTR (red bars) or PIVGFP (green bars) at day 0, and ion transport studies were performed at days specified (*n* = 8 cultures derived from 3 different patients). Significant CFTR function could be measured for up to 8 d but waned by 21 d pi in CF HAE inoculated with PIVCFTR. No significant CFTR function was detected in CF HAE inoculated with PIVGFP. (B) Histological Cytospin assessment of cells shed into apical surface secretions at day 6 pi showing few shed ciliated cells in mock-inoculated CF HAE (i and iii), whereas significant numbers of cells were shed after PIVGFP (ii and iv). Cytospinned apical washes were counterstained with Giemsa (i and ii) or probed with Abs to GFP (green) and acetylated alpha-tubulin (red) to show ciliated cells. Bar represents 20 µm. Images are representative of Cytospins from two individual experiments. (C) Loss of PIV F-positive cells over time after inoculation with PIVCFTR (red bars) or PIVGFP (green bars), showing that PIVCFTR and PIVGFP infected equal numbers of ciliated cells at day 2, but that ciliated cell shedding was delayed for PIVCFTR versus PIVGFP by day 8. Data derived from same dataset in (A). An asterisk (*) denotes *p*<0.05. Error bars indicate SEM.

Duration of CFTR correction is limited by shedding of PIV-infected ciliated cells. Experiments to determine the duration of PIVCFTR-mediated bioelectric correction showed that significant functional correction was maintained for at least 1 wk with detectable, but decreased, levels of function remaining at 21 d pi (Figure 6A). Previously, we have shown that PIV-infected ciliated cells are shed from HAE 3–7 d pi by a poorly understood process of extruding infected ciliated cells from the epithelium onto the lumenal surfaces of HAE [17]. This process likely represents an innate defense function of the epithelium to rid itself of PIV-infected ciliated cells. To confirm that PIV-infected ciliated cells were being shed from the epithelium, we assessed the cellular composition of apical secretions 6 d after PIVGFP inoculation. By morphologic and immunodetection of GFP-positive cells and ciliated cells, we determined that PIV-infected ciliated cells were shed into apical secretions at a rate far exceeding that of natural ciliated cell shedding (Figure 6B). Therefore, the temporally related loss of CFTR functional activity in PIVCFTR-corrected CF HAE likely reflects shedding of infected ciliated cells and suggests that the extent of correction is directly related to the numbers of ciliated cells expressing CFTR. To further explore this relationship, we counted the numbers of PIV-positive cells present in CF HAE over time and show that the loss of PIV-positive ciliated cells paralleled the loss of Cl^−^ transport (compare Figure 6C to 6A), suggesting that the magnitude of correction was indeed directly proportional to the number of CF ciliated cells expressing CFTR.

Interestingly, although the numbers of PIV-positive ciliated cells were similar at day 2 and 4 pi for both transgenes (*CFTR* or *GFP*), by day 8 pi, significantly more PIVCFTR-positive ciliated cells remained compared to PIVGFP. Because PIVCFTR has a lower replication capacity than PIVGFP, we speculate that ciliated cell shedding is related to the rate of virus replication and that identification or generation of PIV vectors with further attenuated replication may provide delivery vectors that prolong functional CFTR correction. Further characterization of the processes involved in PIV-induced ciliated cell shedding from HAE may also provide novel strategies to prolong the lifespan of CFTR-expressing ciliated cells.

### Efficacy of CFTR Correction Is Related to the Number of Surface Epithelial Cells Expressing CFTR

A central question in CF gene transfer studies has been the efficiency of CFTR delivery required for clinical benefit. For chemical corrector therapies, efficiency reflects the percent increase in CFTR function per cell. With respect to gene transfer studies, when CFTR expression exceeds endogenous levels on a per cell basis, it may be speculated that efficiency reflects the number of cells within the epithelium targeted for CFTR delivery. Given the low endogenous level of CFTR expression in ciliated cells, coupled with high levels of exogenous CFTR expression generated from PIV vectors, we performed dose-effect experiments with PIV designed to ask what percentage of CF surface epithelial cells must be corrected to restore hydration and MCT to the airway surface in CF HAE.

**Figure 7:**
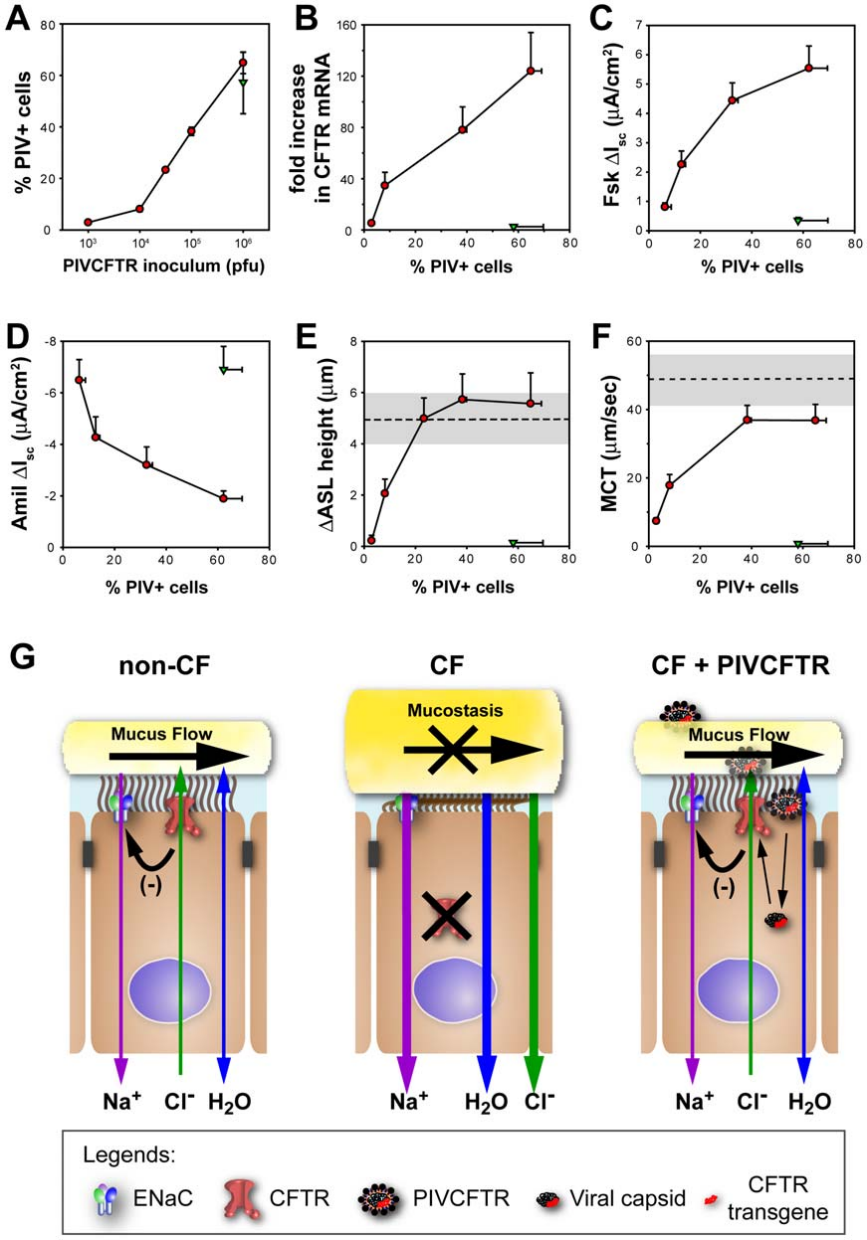
How many CF airway epithelial cells require CFTR to restore ASL homeostasis and MCT to non-CF HAE levels? (A) Quantitation of the percentage of PIV F-positive cells 24 h after inoculation with different concentrations of PIVCFTR (10^3^–10^6^ PFU) and correlation of percentage of PIVCFTR-positive cells to: (B) increased CFTR mRNA expression levels; (C) Fsk-activated changes in *I*_sc_; (D) amil-induced changes in *I*_sc_; (E) ASL height; and (F) MCT rates. All measurements were made 24 h after inoculation with PIVCFTR (closed red circles) or controls (PIVGFP or PIVΔF508CFTR, closed green triangles) as described in Materials and Methods. *n*>11 for each data point representing cultures from 2 or 3 different donors. Dashed lines and grey shaded regions represent mean and standard deviations of ASL heights and MCT rates measured in parallel experiments with non-CF HAE. For these experiments, approximately 80% of surface cells were ciliated cells. (G) Schematic representation depicting the role of CFTR and ENaC in ASL homeostasis in non-CF airway epithelium; the presence of CFTR modulates ENaC activity and combined regulation of these ion channels dictates ASL depth regulation at a level sufficient for effective MCT (black arrow). In CF airway epithelium, the absence of CFTR reduces fluid secretion and leads to dysregulation of ENaC activity overall resulting in hyperabsorption of surface fluid, dehydration of ASL, and mucostasis with accumulation of mucus plugs. Delivery of CFTR to ciliated cells of CF HAE by PIV restores CFTR function, ENaC regulation, ASL homeostasis, and MCT. Error bars indicate SEM.

First, we determined that inoculation of CF HAE with different concentrations of PIVCFTR (range 10^3^–10^6^ PFU) resulted in inoculum-dependent increases in the percentage of PIV-positive cells at 24 h pi (Figure 7A). Increasing numbers of cells expressing CFTR paralleled increasing *CFTR* mRNA levels, Fsk-stimulated Cl^−^ secretion, and Amil-sensitive Na^+^ absorption (Figure 7B, 7C, and 7D).

We next used this approach to determine the percentage of cells required to express CFTR to restore normal ASL homeostasis and MCT to CF HAE under thin-film conditions. ASL height and MCT were measured in CF HAE 24 h after inoculation with PIVCFTR at different concentrations. PIVCFTR resulted in concurrent increases in ASL height and MCT rates that were proportional to the percentages of PIV-positive cells, with a plateau occurring at approximately 40% of PIV-positive cells (Figure 7E and 7F). As controls, PIV expressing GFP or *ΔF508CFTR* failed to increase ASL height or MCT rates in CF HAE (Figures 7E and 7F). By comparison of these data to ASL height and MCT measurements in non-CF HAE (Figure 7E and 7F, dashed lines), it was noted that ASL height and MCT rates both plateaued at levels similar to those measured in non-CF HAE, suggesting that normal homeostasis had been reached. We speculate that the plateau levels for ASL height and MCT reflect homeostatic feedback signals within ASL under the thin-film conditions, e.g., ATP release rates that regulate ion channel activity.

By comparison of data obtained in corrected CF HAE to that in normal non-CF HAE, we calculate that at least 25% of surface epithelial cells (30% of ciliated cells) required CFTR expression to restore ASL height regulation to non-CF HAE levels. For MCT, approximately 40% of cells (50% of ciliated cells) required CFTR expression to approach MCT rates measured in parallel non-CF HAE. These data identify the efficiency of epithelial cell CFTR delivery to restore defective MCT in CF HAE. Of note is the observation that although PIVCFTR was able to restore MCT to nearly normal levels, MCT, unlike ASL height, was not fully restored. This discrepancy is likely related to the cytotoxic effects of PIV infection on MCT, but not ASL measurements. In these experiments, performed 24 h after inoculation, MCT restoration is much improved over that measured at 48 h pi (Figure 5D), further suggesting that 48 h, but not 24 h, of infection with this prototypic PIV vector had detrimental effects on MCT. These data, taken together with those indicating that overexpression of CFTR on a per cell basis was not detrimental to ion and fluid transport processes, indicate that gene delivery vectors capable of targeting at least 25% of the surface airway epithelial cells will be sufficient to restore ASL height regulation and MCT to levels comparable to those exhibited in non-CF airway epithelia and that precise regulation of CFTR levels at least in ciliated cells is not required.

## Discussion

Successful gene transfer to CF airways in vivo has been principally hampered by a lack of efficacy due to the inefficiency of gene transfer to human airway epithelium that normally exhibits CFTR function [30]–[33]. Although there is clear evidence that both airway surface and submucosal gland epithelia are dysfunctional in CF, presently, the precise airway regions of the CF lung that require CFTR delivery for restoration of normal physiological function and reduction of disease symptoms are not well established. Although dependent on antibodies used, CFTR has been localized to human ciliated cells [3] and the fluid-secreting cells of the submucosal glands [4]. Previously noted physiologic characteristics of ciliated cells also indicate that ciliated cells function to maintain airway surface hydration [34]. Certainly, ciliated cells facilitate effective MCT and airway mucus clearance. Together, these properties of ciliated cells combined with the abundance of ciliated cells throughout the human airways make this cell type a logical, although not exclusive, target for CF lung gene delivery strategies.

In this study, we have shown that PIV-mediated CFTR delivery to ciliated cells is efficient and sufficient for correcting the CF airway epithelium phenotype, i.e., efficient delivery of CFTR to CF human ciliated airway epithelium corrected hallmark characteristics of CF HAE that mimic the initiating events of CF lung disease, i.e., abnormal ASL volume homeostasis and mucostasis (Figures 5 and 7). Abnormal ASL homeostasis in CF airway epithelium is due to dysregulated Na^+^ and Cl^−^ ion transport [35], both consequent to the absence/dysfunction of CFTR at the apical membrane [24]. Here, we have demonstrated that delivery of CFTR to ciliated cells restores Cl^−^ secretion and reduces the Na^+^ hyperabsorption characteristic of the CF airway epithelium in vitro and in vivo, providing confirmatory evidence that CFTR functions as both a Cl^−^ channel and regulator of ENaC within ciliated cells. Further, we have demonstrated that correction of the ion channel defects of CF HAE restores the integrated physiology required for ASL regulation, which ultimately restores MCT (shown schematically in Figure 7G).

A critical variable for restoration of CFTR functional activity by PIV is the percentage of cells expressing CFTR. Using CF HAE, we demonstrate that restoration of normal ASL height and MCT required CFTR delivery to approximately 25% and approximately 40% of surface epithelial cells, respectively. We suggest that restoration of ASL height is the most predictive measure for these studies, as PIV clearly exerts cytotoxic effects on MCT, but not ASL measurements. These effects were isolated to MCT, but not ASL, suggesting that virus-mediated cytotoxicity may affect the synchrony of cilia beat, leading to modestly reduced effectiveness of ciliated cells to transport mucus.

Previous studies with gap junction–coupled polarized, but not differentiated, airway epithelial cell lines suggested that approximately 6%–10% of cells required CFTR to correct the Cl^−^ transport defect [36], whereas almost all cells (>90%) required CFTR overexpression to correct ENaC hyperabsorption [21]. Clearly, expression of CFTR in nonciliated airway epithelial cells would be predicted to increase fluid secretion onto the apical surfaces of these cells although these previously published studies did not test this hypothesis. In our studies, we have directly shown that expression of CFTR in 60% of CF ciliated cells fully corrects the ENaC hyperabsorption defect (Figure 5A) and that CFTR expression in approximately 25% of cells (approximately 30% of ciliated cells) corrects ASL volume homeostasis in CF HAE (Figure 7E). With respect to ENaC activity after CFTR delivery, the reasons why our data differ from these previous studies are unclear, but it may be speculated that differentiated human airway epithelium models as used in this current study are more representative and relevant to the required efficiency of CFTR delivery to human airway epithelium in vivo.

Although our data indicate that CFTR delivery to CF ciliated cells is sufficient for restoring MCT to CF HAE, it is likely that delivery of CFTR to other nonciliated surface epithelial cells may provide functional CFTR activity capable of hydrating the airway surface. At present, we are not aware of gene delivery vectors capable of delivering CFTR exclusively to nonciliated cells of HAE to determine whether CFTR expression in nonciliated cells also restores MCT. In our studies, we have combined the requirement of ciliated cells for generation of MCT with CFTR targeting of CF ciliated cells to restore defective MCT. Since ciliated cells are the predominant airway surface epithelial cell type throughout the human conducting airways, vectors targeting at least ciliated cells may achieve the required efficiency of delivery for restoration of MCT. We propose that targeting at least ciliated cells provides efficient and effective CFTR function that is sufficient for restoration of MCT.

Our data using an in vitro model of human airway epithelium predict that CFTR delivery to 25% of CF airway epithelial cells will restore MCT to near normal levels. However, it remains to be determined what proportion of normal MCT rates in vivo would be beneficial to CF patients. Tracheal mucus velocities in young smokers are significantly reduced compared to young nonsmokers (3.4 mm/min versus 10.0 mm/min) but without significant differences in lung function, perhaps suggesting that MCT at rates below “normal” may be sufficient to maintain pulmonary healthy [37]. These in vivo studies measured mucus velocities only in the trachea, whereas CF lung disease likely initiates in the more vulnerable, smaller bronchiolar airway regions. If these regions respond similarly to CFTR delivery, then it is possible that delivering CFTR to fewer than 25% of CF cells may provide sufficient MCT to maintain healthy airways. Further testing of this hypothesis will require appropriate in vivo studies.

Since PIV infects ciliated airway epithelium of hamsters and human and nonhuman primates, but not those of the murine airways, testing our PIV vectors in vivo in appropriate models is difficult. Additionally, it has been recently reported that expression of human or murine CFTR in ciliated cells of *CFTR^−/−^* mice failed to correct the nasal epithelium bioelectric defect, although correction was demonstrated in neonatal, but not adult, tracheal epithelium [38]. One explanation for these results, in contrast to our data, is that murine CFTR expressed in murine ciliated cells may not function correctly. Our study highlights the need to test vectors for CFTR delivery in appropriate human models and that such data obtained from CF mouse models require cautious interpretation. The recent generation of a CF pig model [39],[40] may be beneficial for testing such vector systems, but preliminary data using ciliated airway epithelial cultures derived from porcine trachea suggest that this species is also not infected by the human viruses from which our PIV vectors are generated.

Using our prototypic PIV vector, we could not determine the lowest limit of CFTR expression on a per cell basis required for correction since this vector significantly overexpressed CFTR relative to endogenous levels. In this regard, because CFTR is critical for regulation of ASL homeostasis, there has been concern that overexpression of CFTR in CF airways would “supercorrect” Cl^−^ transport and generate excessive fluid secretion. This concern, while reasonable, appears unwarranted based on our observations that ion transport rates and ASL heights in CF HAE after CFTR delivery did not exceed that measured in non-CF HAE (Figures 3, 5, and 7). These data agree with previously published reports in which CFTR was transgenically overexpressed in mouse airway epithelium without deleterious results in terms of cell or organ toxicity [22]. However, in this transgenic study, CFTR was overexpressed in Clara cells and alveolar type II cells of the mouse lung, and so our study represents the first demonstration, to our knowledge, of the functional safety of CFTR overexpression in human ciliated cells. Since CFTR overexpression did not supercorrect ASL regulation, we speculate that normal airway epithelium exhibits multiple apical membrane regulatory mechanisms in addition to CFTR levels that prevent excessive secretion of fluid into the airway lumen, i.e., airway flooding.

Demonstrating that PIV expresses CFTR at levels in excess of those required to restore full function to CF HAE suggests that further attenuation of PIV will be feasible while still providing sufficient CFTR for functional correction. Indeed, the lower replication capacity 10-fold reduction of PIVCFTR compared to PIVGFP, in the context of >100-fold overexpression of CFTR in individual ciliated cells, suggests that further attenuation of PIV will continue to provide sufficient CFTR for correction of the CF MCT defect and possibly further reduce the generation of inflammatory mediators and cytotoxicity associated with our PIV vector prototype. The continued effort to develop vaccines against PIV has generated a wealth of live attenuated recombinant PIV [41] that exhibit attenuated replication. It is interesting to note that PIV3 vaccine candidates have been extensively evaluated after lumenal airway delivery in adults and infants as young as 3 mo [42]; an age of CF patients in which CFTR replacement would be desirable.

The demonstration of efficacious *CFTR* gene delivery to human ciliated airway epithelium overcomes a major hurdle to gene transfer approaches for CF lung disease. Other strategies to improve gene delivery to the human airways are ongoing and are focused on vector development [43]–[45] and/or manipulation of the host tissue [46],[47]. However, the results so far published have not shown significant improvement in the ability to deliver transgenes to human ciliated airway epithelium. Lentiviral-based vectors pseudotyped with Ebola, influenza virus, baculovirus, Sendai, or SARS-CoV envelope proteins efficiently transduce airway epithelial cells in vitro and murine airways in vivo [48]–[53], suggesting that combining useful envelope glycoproteins with the potential longer duration of gene expression afforded by lentiviruses may provide novel vectors for lung gene transfer strategies. Similar vectors can be envisioned using the glycoproteins of PIV to target lentiviruses to human ciliated airways. However, to date, none of these vector systems have progressed to functional studies after delivery of CFTR to human ciliated airway epithelium, and no demonstration of correction of the CF phenotype (e.g., ASL height or MCT) has been reported.

Collectively, the studies reported here demonstrate the efficiency of CFTR delivery to human CF ciliated airway epithelium that is sufficient to reverse the CF phenotype of ASL dehydration and mucostasis. Our prototypic PIV vector provides a useful tool for manipulating ciliated cell function and for investigating the future potential of delivering functional CFTR to the airways of CF patients.

## Materials and Methods

### Construction of PIVCFTR, PIVΔF508CFTR, and PIVGFPCFTR

Recombinant hPIV3 (NC_001796) encoding human *CFTR* (NM_000492) or *ΔF508CFTR* cDNA or *GFP* and *CFTR* as separate genes was generated from the cDNA antigenome of full-length hPIV3 JS strain and described in detail in the Supplemental Methods (Text S1). After rescue, PIV replicated in HEp2 cells to a titer comparable to the JS wild-type strain (10^9.1^ 50% tissue culture infective dose [TCID_50_]/ml) suggesting that GFP, *CFTR*, or *ΔF508CFTR* did not adversely affect the growth capacity of PIV in producer epithelial cell lines. Virus titers generated by CF HAE were determined in duplicate by procedures previously described [17].

### Human Airway Epithelial Cell Cultures

Human tracheobronchial tissues were obtained by the University of North Carolina (UNC) CF Center Tissue Culture Core from airways resected from CF (*ΔF508/ΔF508* mutation) or non-CF patients undergoing elective surgery under UNC Institutional Review Board–approved protocols. Isolated epithelial cells were obtained and plated at a density of 250,000 cells per well on permeable Transwell-Col supports (T-Col, 12-mm diameter; Corning-Costar [13],[54]). For bioelectric measurements in Ussing chambers, cells were plated on type IV collagen-coated Snapwell supports (Corning-Costar). HAE were generated by provision of an air–liquid interface for 4–6 wk to form well-differentiated, polarized cultures that resemble in vivo pseudostratified ciliated epithelium [13]. Prior to viral inoculation, the apical surfaces of HAE were rinsed three times over 15 min and inoculated with 100 µl of 10^7^ PFU/ml virus stocks for 2 h at 37°C (unless otherwise described). After removal of inoculum, HAE were returned to humidified incubators. Human tracheobronchial tissues with 1 cm^2^ of epithelial cell surface area were inoculated with 100 µl of PIVGFP (10^7^ PFU/ml) or vehicle control for 2 h at 37°C, and then tissues were washed in medium and returned to the incubator for 24 h in minimal media volume. After fixation in 4% PFA, tissues were paraffin-embedded, and histological sections were prepared. Immunodetection of GFP was performed as described below.

### Immunodetection Protocols

To determine epithelial cell types infected by PIVGFP in vitro, HAE were fixed in 4% PFA, permeabilized with 1% Triton X-100, and ciliated cells identified with mouse primary antibodies (Ab) against acetylated α-tubulin (Zymed Laboratories) and anti-mouse IgG-Texas Red (Jackson ImmunoResearch) using confocal X-Z scanning microscopy. Ex vivo tissues were paraffin-embedded, and histological sections were prepared. GFP signal was enhanced by indirect immunofluorescence with rabbit anti-GFP polyclonal Ab (Ab-Cam) and goat anti-rabbit IgG-fluorescein (Jackson ImmunoResearch). Both in vitro and ex vivo, GFP colocalized to cells that were also positive for acetylated α-tubulin, confirming the targeting of ciliated cells in vitro and ex vivo by PIV.

Immunolocalization of PIV F protein was performed on HAE fixed in 4% PFA and immunostained either en face or on paraffin-embedded histological sections. For en face detection, the apical surfaces of HAE were incubated with PIV3 F-specific monoclonal Ab (clone 216.16), followed by goat anti-mouse IgG-AlexaFluor594 (Invitrogen). Quantitation of percentages of cells expressing PIV F protein was performed as described previously [17] by assessing four different en face fields of the HAE surface with two cultures obtained from each of three patients. For histological sections, HAE were immunostained as previously described [17]. GFP fluorescence was enhanced as described above. To immunolocalize CFTR in ciliated cells, CF and non-CF HAE inoculated with PIVGFPCFTR or PIVGFP were gently scraped with pipette tips and cell suspensions in PBS, immediately pelleted onto glass slides with Cytospin, and then air-dried. Following fixation in 4% PFA, CFTR was detected with anti-human CFTR mouse monoclonal Ab #596 (a gift from Dr. J. Riordan, University of North Carolina at Chapel Hill) and Alexafluor594-conjugated goat anti-mouse antibody. Cell nuclei were counterstained with Hoechst 33342 (Invitrogen). Images were taken with a Leica SP2 Laser Scanning Confocal Microscope, and processed with Adobe Photoshop CS2.

To assess ciliated cell shedding induced by PIV, the apical surfaces of CF HAE inoculated with PIVGFP or mock were washed in 200 µl of PBS for 30 min, harvested, and washes pelleted onto glass slides using a StatSpin Cytofuge2 (Iris Sample Processing) and then air-dried. Slides were then counterstained with Giemsa (Invitrogen) or probed with rabbit anti-GFP polyclonal Ab (Ab-Cam) with goat anti-rabbit IgG-fluorescein (Jackson ImmunoResearch) and Ab against acetylated α-tubulin (Zymed Laboratories) with anti-mouse IgG-Texas Red (Jackson ImmunoResearch). Cell nuclei were counterstained with Hoechst 33342 (Invitrogen). Fluorescent confocal images and DIC were taken with Leica SP2 Laser Scanning Confocal Microscope. Image processing and overlay were done with Adobe Photoshop CS2.

Western blot analyses of CFTR protein was performed on HAE lysed in M-PER buffer (Pierce). Equal amounts of total protein (850 µg) per sample were adjusted to 1 ml volume with lysis buffer and added to 2 µl of anti-CFTR Ab #596, followed by 50 µl of immobilized-protein G agarose bead slurry (Pierce). Proteins were released from beads with sample buffer, separated with a NuPAGE 3%–8% Tris-Acetate Gel (Invitrogen), and transferred to PVDF membranes. The membranes were then incubated with anti-CFTR Ab (#596) followed by goat anti-mouse IgG-HRP (Jackson ImmunoResearch), and CFTR were visualized with SuperSignal West Dura Substrate (Pierce).

### Quantitative Reverse Transcriptase PCR for CFTR

Total RNA was isolated HAE after inoculation with either PIVCFTR, PIVGFP, or vehicle alone using acid phenol-guanidine thiocyanate followed by DNase digestion and further purification using the Qiagen RNeasy Mini Kit. RNA from three individual CF HAE per inoculation was pooled and first-strand cDNA was synthesized with oligo(dT) and SuperScript II reverse transcriptase (Invitrogen) to ensure amplification of mRNA and not viral genome RNA. Quantitative PCR was performed using a Roche LightCycler with the Roche FastStart DNA Master SYBR Green I Kit according to the manufacturer's protocols. Using the LightCycler Software version 4.0, levels of *CFTR* mRNA were normalized to the level of GAPDH.

### Inflammatory Mediator Measurements

Apical and basolateral samples were collected 48 h pi by applying 0.2 ml of serum-free medium to apical surfaces and harvested 30 min later. Basolateral samples were harvested from the basolateral medium. Samples were stored at −80°C before cytokine analyses using 28-plex Beadlyte Assays (Upstate) with Luminex technology (see Text S1 for details).

### Ion Transport Measurements

HAE were mounted in Ussing chambers for measurement of transepithelial resistance (*R_t_*), transepithelial potential difference (*V_t_*), and short-circuit current (*I*_sc_) as previously described [55]. HAE were bathed in bilateral Krebs Bicarbonate Ringer solution (KBR) gassed with 95% O_2_, 5% CO_2_, and maintained at 37°C. *V_t_* was clamped to zero, and pulsed to ±10 mV for 0.5 s every 60 s. The electrometer output was digitized online, and *I*_sc_, *R_t_*, and calculated *V_t_* displayed on a video monitor. Drugs (amiloride [10^−5^ M], forskolin [10^−6^ M], and UTP [10^−4^ M]) were added from concentrated stock solutions to either lumenal and/or serosal surfaces (all obtained from Sigma-Aldrich). CFTR_172_ (10^−5^ M) was synthesized from a local source according to appropriate standards and used as previously described [20]. CFTR_172_ was added to the apical or basolateral bath 15 min before or during forskolin-activated CFTR ion transport. For the basolateral studies, CFTR_172_ did not affect forskolin-activated responses even when left 15 min until addition of CFTR_172_ to the contralateral surface. For the time course of CFTR functional activity experiments, all CF HAE were inoculated with either PIVGFP or PIVCFTR at day 0, and on specific days, cultures were mounted in Ussing chambers for analyses.

For microelectrode measurements of *V_t_* in thin films of ASL, borosilicate glass microelectrodes (World Precision Instruments) were filled with 3 M KCl and positioned into the ASL by a motorized micromanipulator (MC1000e; SD Instruments) connected to a high-impedance electrometer (World Precision Instruments). A macroelectrode, constructed of polyethylene tubing containing 3 M KCl/4% agar, was placed in the serosal bath as the ground. To measure the contribution of basal Cl^−^ and Na^+^ transport to *V_t_*, bumetanide (10^−4^ M) and benzamil (10^−5^ M), respectively, were added to the basolateral bath 10 min prior to recording, as previously described [35]. To avoid evaporation of the thin ASL layer in low-humidity environments, 100 µl of immiscible perfluorocarbon (Fluorinert-77; 3 M Corporation) was added to the airway surface as previously described [56].

### ASL Height Measurements

To visualize the ASL height, 25 µl of PBS containing 0.2% vol/vol Texas Red-dextran (10 kDa; Invitrogen) was added to the lumenal surfaces of HAE. This volume of PBS results in an initial ASL height of approximately 20–30 µm, as previously described [56]. Images of the Texas Red–labeled ASL are acquired by laser-scanning confocal microscopy (Zeiss Model 510) using the appropriate filters, 540 nm excitation/630 nm emission for Texas Red. Perfluorocarbon was added to the airway surface 10 min after the addition of the dye to avoid evaporation of ASL as described above. ASL height was determined by averaging the height obtained from *XZ* scans of five predetermined points per HAE over time [56].

### Cilia Beat Frequency Measurements

HAE were rinsed three times with PBS, then placed on an inverted phase contrast microscope (TE 2000; Nikon) to record cilial movement with a 20× objective. High-speed (125 Hz) video images were captured with an eight-bit b/w camera (GS-310 Turbo; Megaplus). The analog signal was digitized via an analog-to-digital converter board (A/D; National Instruments). A digital computerized CBF analysis system was used to analyze the acquired video images, using specialized software, based on Sisson-Ammons Video Analysis [57].

### Measurement of MCT Rates

HAE were removed from a well-humidified incubator and washed three times with PBS, and green fluorescent microspheres (0.02% vol/vol, 1 µm; Invitrogen) were added to apical surfaces in 20 µl of PBS and then HAE immediately returned to the incubator. The rate of microsphere displacement was measured from time-lapse fluorescent images (488 nm excitation/530 nm emission) acquired for 3 s with an inverted epifluorescence microscope (Eclipse; Nikon) and a charge-coupled device (CCD) camera (OrcaER; Hamamatsu). Angular bead transport velocity was calculated as previously described [6].

### Statistics

All data are expressed as means±standard error of the mean (SEM) and followed normal distribution as assessed by a standard Normality Test (Kolmogorov-Smirnov). Unpaired Student *t*-test was used to assess the difference between groups. One-way ANOVA was performed when more than two groups were compared with a single control, and then the differences between individual groups within the set assessed by a multiple-comparison test (Tukey) when the *F* was <0.05. A *p*-value of <0.05 was considered significant.

## Supporting Information

Figure S1 **Schematic representation of construction of PIVCFTR, PIVΔF508CFTR, and PIVGFPCFTR.** The coding sequence for CFTR was inserted into the downstream noncoding region of the *HN* gene. Nucleotides 8598–8603 of the PIV3 genome were modified into a StuI site, which was used to accept a linker that contained the PIV3 gene-end (GE), intergenic (IG), and gene-start (GS) transcription signals, followed by SacII and ApaI sites. These latter sites were used to accept a SacII-ApaI fragment containing the open reading frame for CFTR (shaded rectangle, with ATG and TAG initiation and termination codons indicated). PIVGFPCFTR was constructed with the same strategy as for PIVCFTR, except with the PIVGFP backbone. Thus, PIVGFP-CFTR expresses two transgenes, *GFP* and *CFTR*, simultaneously.
(0.41 MB TIF)

Figure S2 **Inflammatory mediators secreted by CF HAE in response to PIVGFP or PIVCFTR.** Luminex bead-based quantitation of inflammatory mediators secreted into apical (positive of abscissa) and basolateral (negative of abscissa) compartments of CF HAE 48 h pi with mock (vehicle control alone) (black bars), UV-inactivated PIVGFP (hatched bars), PIVGFP (green bars), or PIVCFTR (red bars). CXCL-10, IL-6, IL-12p40, MCP-1, RANTES, and CXCL-8 were the only analytes of 27 tested that were significantly altered by virus infection. In all cases, PIVCFTR infection resulted in decreased secretion of inflammatory mediators compared to PIVGFP and reducing secretion to that measured after UV-PIVGFP alone (*n* = 4 for each point, *ns* denotes not statistically significant differences).
(0.52 MB TIF)

Figure S3 **PIV Infection and CFTR is only detected in ciliated cells.** CFTR or PIV antigens were immunodetected with anti-CFTR # 596 (red) or rabbit polyclonal anti-PIV (green), respectively. (A) CFTR was detected in PIVCFTR-inoculated non-CF ciliated cells as well as noninoculated ciliated cells. (B) In CF HAE, PIVCFTR only infected ciliated cells and expressed transduced CFTR. No CFTR or PIV antigen was detected in nonciliated cells (arrowheads). (C) Endogenous CFTR was only detected in ciliated cells from non-CF HAE. For (B and C), arrowheads show absence of CFTR in nonciliated cells. Bar represents 10 µm.
(6.24 MB TIF)

Figure S4 **Effect of PIV-mediated CFTR expression in CF HAE on UTP-mediated Cl^−^ secretion and transepithelial resistance.** Changes in UTP-mediated *I*_sc_ responses (hatched bars, left ordinate) and transepithelial resistance (grey bars, right ordinate) 48 h pi with vehicle alone, PIVGFP, or PIVCFTR. PIV infection of CF HAE resulted in a potentiated UTP response over vehicle control but did not significantly affect transepithelial resistances.
(0.37 MB TIF)

Text S1 **Additional methodologies are described.** These include the detailed procedures for the molecular constructions of recombinant PIV vectors (PIVCFTR, PIVΔF508CFTR, and PIVGFPCFTR), the oligonucleotide sequences for primers used for qRT-PCR assays, and the list of inflammatory mediators measured by Luminex multiplex assays.
(0.03 MB DOC)

## Abbreviations

Ab: antibody
ASL: airway surface liquid
CBF: ciliary beat frequency
CF: cystic fibrosis
CF HAE: human ciliated airway epithelial cell cultures from cystic fibrosis patient airways
ENaC: epithelial sodium ion channel
*I*_sc_: short-circuit current
KBR: Krebs buffered Ringer bathing solution
MCT: mucus transport
PFU: plaque-forming unit
pi: postinoculation
PIV: parainfluenza virus
*V_t_*: transepithelial potential difference
