# Supplementary figures and images for: CFTR Delivery to 25% of Surface Epithelial Cells Restores Normal Rates of Mucus Transport to Human Cystic Fibrosis Airway Epithelium
Source: PLoS Biol. 2009 Jul 21;7(7):e1000155. doi: 10.1371/journal.pbio.1000155 (PMC2705187; doi:10.1371/journal.pbio.1000155)

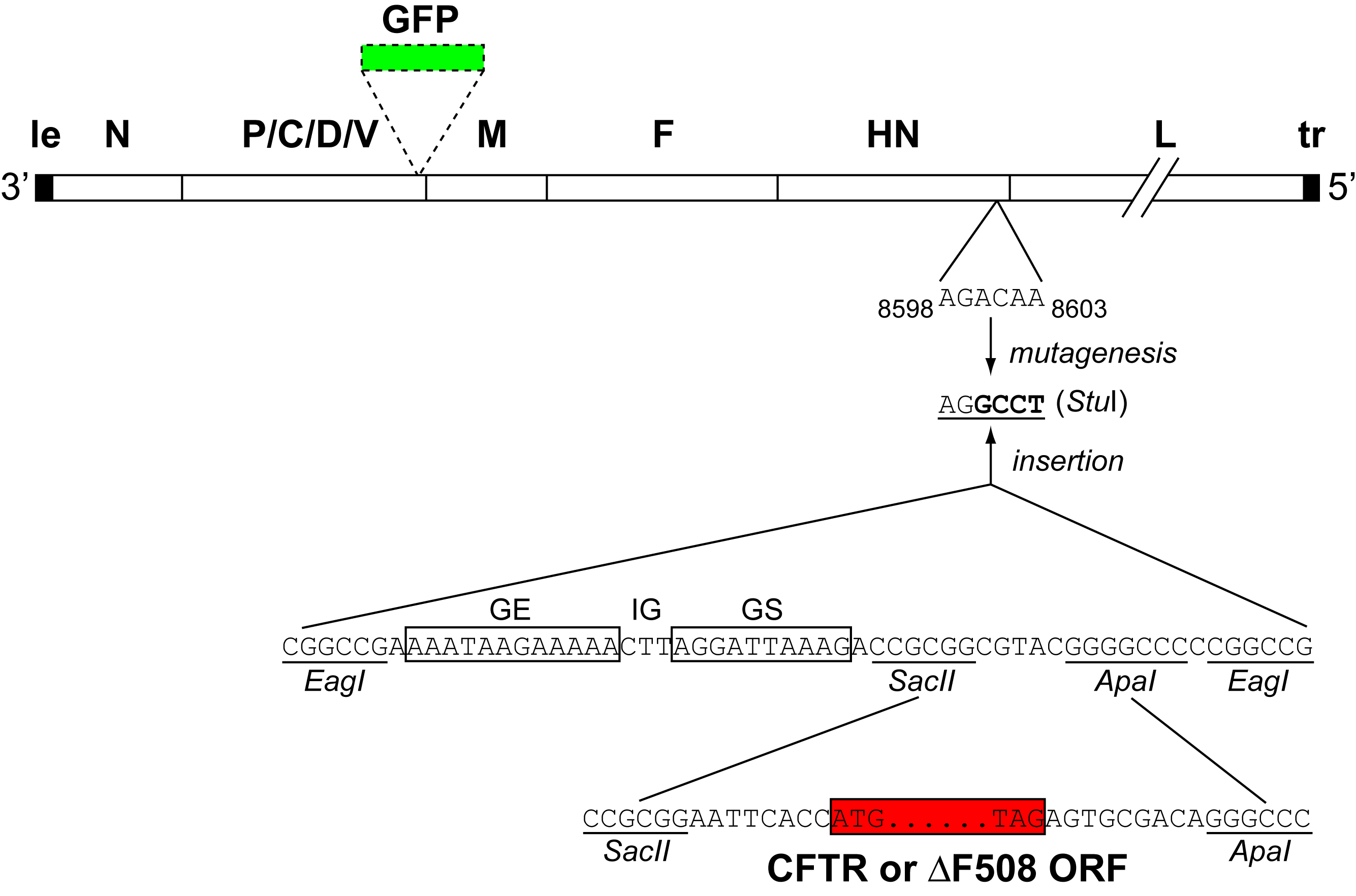

Supplement: Figure S1 — Schematic representation of construction of PIVCFTR, PIVΔF508CFTR, and PIVGFPCFTR. The coding sequence for CFTR was inserted into the downstream noncoding region of the HN gene. Nucleotides 8598–8603 of the PIV3 genome were modified into a StuI site, which was used to accept a linker that contained the PIV3 gene-end (GE), intergenic (IG), and gene-start (GS) transcription signals, followed by SacII and ApaI sites. These latter sites were used to accept a SacII-ApaI fragment containing the open reading frame for CFTR (shaded rectangle, with ATG and TAG initiation and termination codons indicated). PIVGFPCFTR was constructed with the same strategy as for PIVCFTR, except with the PIVGFP backbone. Thus, PIVGFP-CFTR expresses two transgenes, GFP and CFTR, simultaneously. (0.41 MB TIF) [file pbio.1000155.s001.tif]

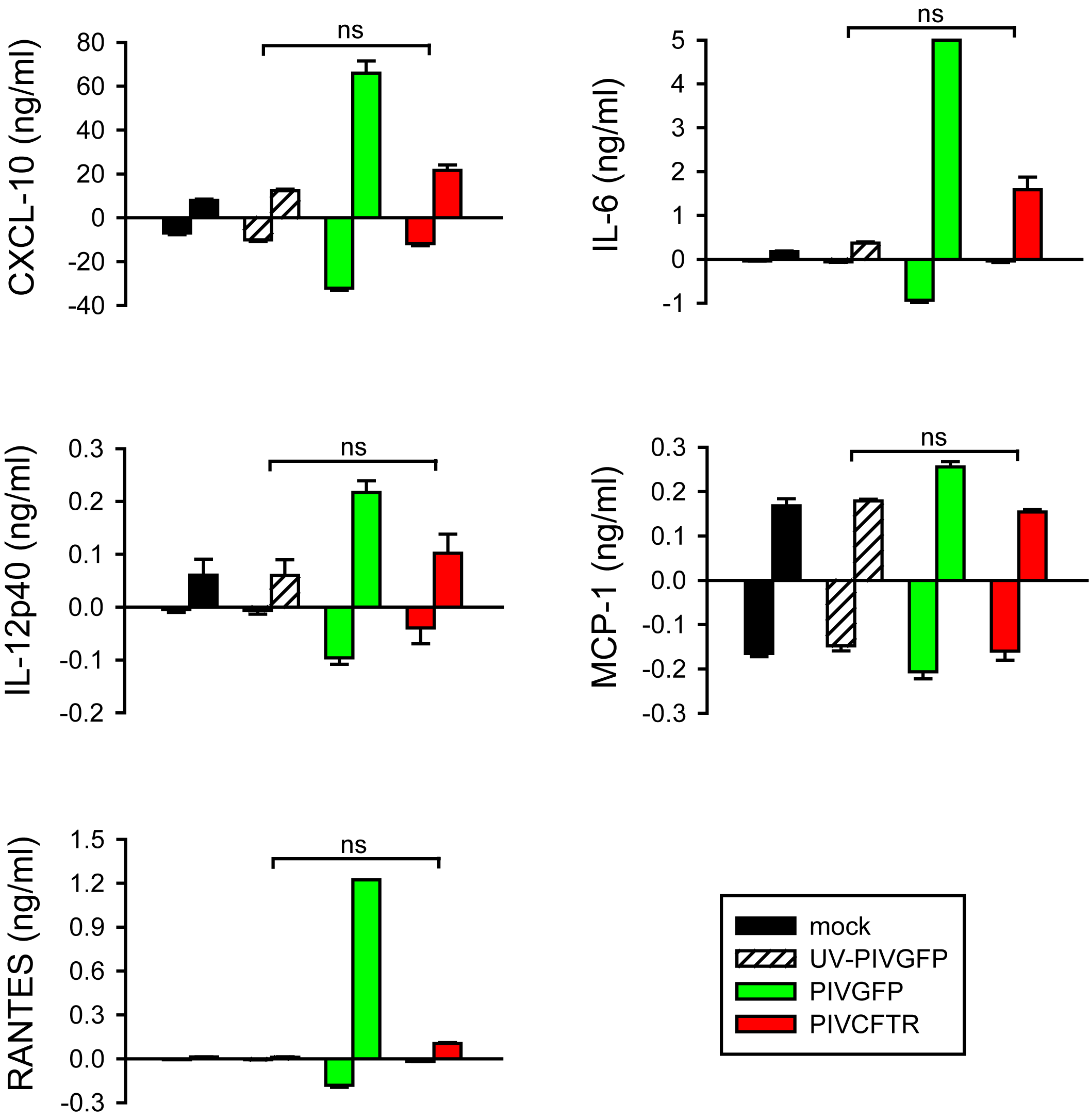

Supplement: Figure S2 — Inflammatory mediators secreted by CF HAE in response to PIVGFP or PIVCFTR. Luminex bead-based quantitation of inflammatory mediators secreted into apical (positive of abscissa) and basolateral (negative of abscissa) compartments of CF HAE 48 h pi with mock (vehicle control alone) (black bars), UV-inactivated PIVGFP (hatched bars), PIVGFP (green bars), or PIVCFTR (red bars). CXCL-10, IL-6, IL-12p40, MCP-1, RANTES, and CXCL-8 were the only analytes of 27 tested that were significantly altered by virus infection. In all cases, PIVCFTR infection resulted in decreased secretion of inflammatory mediators compared to PIVGFP and reducing secretion to that measured after UV-PIVGFP alone (n = 4 for each point, ns denotes not statistically significant differences). (0.52 MB TIF) [file pbio.1000155.s002.tif]

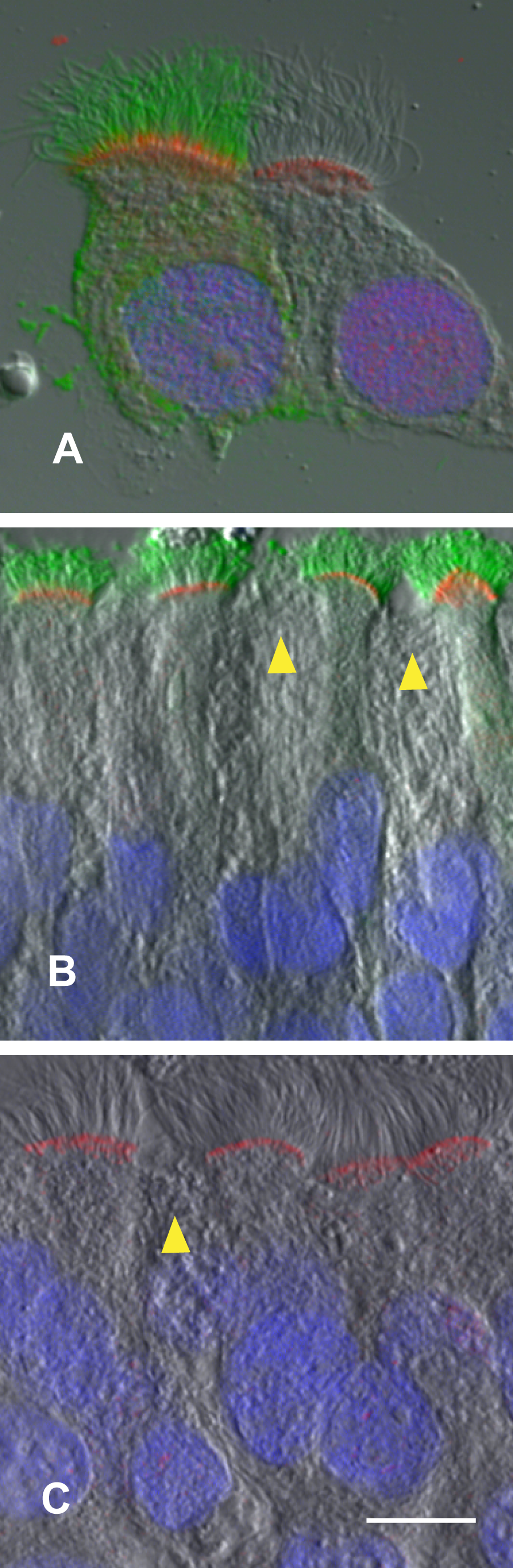

Supplement: Figure S3 — PIV Infection and CFTR is only detected in ciliated cells. CFTR or PIV antigens were immunodetected with anti-CFTR # 596 (red) or rabbit polyclonal anti-PIV (green), respectively. (A) CFTR was detected in PIVCFTR-inoculated non-CF ciliated cells as well as noninoculated ciliated cells. (B) In CF HAE, PIVCFTR only infected ciliated cells and expressed transduced CFTR. No CFTR or PIV antigen was detected in nonciliated cells (arrowheads). (C) Endogenous CFTR was only detected in ciliated cells from non-CF HAE. For (B and C), arrowheads show absence of CFTR in nonciliated cells. Bar represents 10 µm. (6.24 MB TIF) [file pbio.1000155.s003.tif]

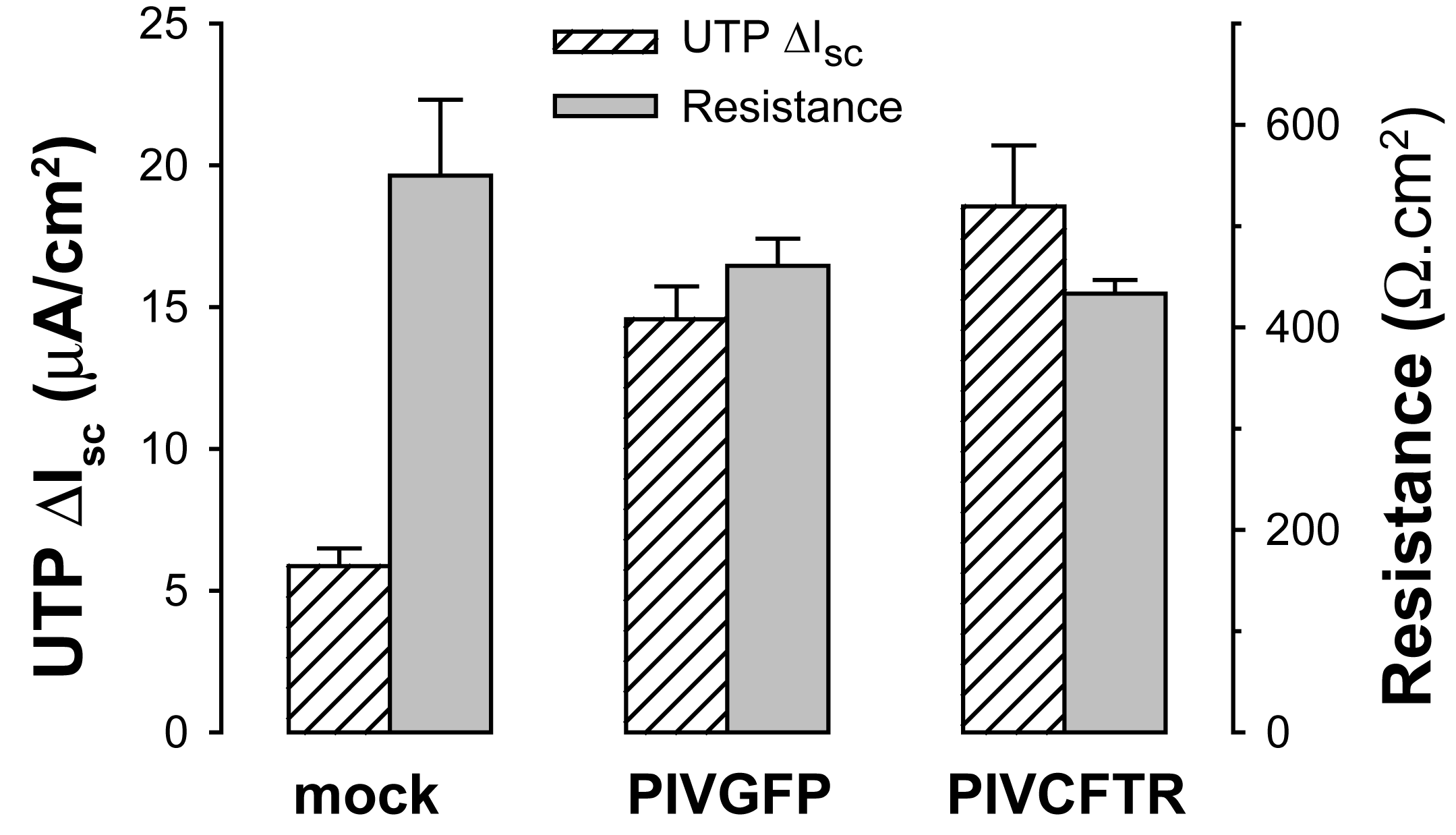

Supplement: Figure S4 — Effect of PIV-mediated CFTR expression in CF HAE on UTP-mediated Cl− secretion and transepithelial resistance. Changes in UTP-mediated I sc responses (hatched bars, left ordinate) and transepithelial resistance (grey bars, right ordinate) 48 h pi with vehicle alone, PIVGFP, or PIVCFTR. PIV infection of CF HAE resulted in a potentiated UTP response over vehicle control but did not significantly affect transepithelial resistances. (0.37 MB TIF) [file pbio.1000155.s004.tif]
