# Supplementary material for: CFTR Delivery to 25% of Surface Epithelial Cells Restores Normal Rates of Mucus Transport to Human Cystic Fibrosis Airway Epithelium
Source: PLoS Biol. 2009 Jul 21;7(7):e1000155. doi: 10.1371/journal.pbio.1000155 (PMC2705187; doi:10.1371/journal.pbio.1000155)
Supplement: Text S1 — Additional methodologies are described. These include the detailed procedures for the molecular constructions of recombinant PIV vectors (PIVCFTR, PIVΔF508CFTR, and PIVGFPCFTR), the oligonucleotide sequences for primers used for qRT-PCR assays, and the list of inflammatory mediators measured by Luminex multiplex assays. (0.03 MB DOC) [file pbio.1000155.s005.doc]

**SUPPLEMENTAL METHODS**

**Construction of PIVCFTR, PIVΔF508CFTR and PIVGFPCFTR**

Nucleotides AGACAA at positions 8598-8603 located in the downstream noncoding region of the HN gene of the wildtype PIV3 genome or PIV3GFP were mutated by PCR into AGGCCT, a *Stu*I restriction site (**Figure S1**). *Stu*I digestion produced blunt ends, with which ligated an oligo-linker containing hPIV3 consensus gene end (GE), inter-genic (IG), gene start (GS) sequences, and restriction sites for *Eag*I (on both ends), *Sac*II, and *Apa*I (CGGCCGAAAATAAGAAAAACTTAGGATTAAAGACCGCGGCGTACGGGGCCCCCGGCCG; *Eag*I sites are underlined). Human CFTR cDNA was PCR-modified to contain *Sac*II and *Apa*I at the 5′ and 3′ termini respectively of the complete CFTR ORF. The PCR product was digested with *Sac*II and *Apa*I, and inserted into the *Sac*II-*Apa*I window of a subclone containing the linker mentioned above. This construct was then digested with *Eag*I and inserted between the *Eag*I-*Eag*I window in the modified hPIV3 anti-genome. In this arrangement, CFTR is flanked by a set of hPIV3 GS and GE signals and expressed as an extra gene product. In PIVCFTR a total number of 4512 nucleotides, equivalent of 29.2% of the wild-type PIV3 genome, were added, complying with the “rule of six” required for this virus type.

**Primers for CFTR quantitative RT-PCR**

Primers for CFTR were 5'-TGACACACTCAGTTAACCAAGGTCAG-3' and 5'-CCTCTGAAGAATCCCATAGCAAGCAA-3', which correspond to Exons 14 and 17 respectively. Primers for GAPDH were 5'-gaaggtgaaggtcggagtca-3' and 5'-gatctcgctcctggaagatg-3'.

**Inflammatory Mediator measurements**

We assessed samples from CF HAE inoculated with vehicle alone, ultraviolet-light inactivated PIVGFP, PIVGFP and PIVCFTR for the following inflammatory mediators using Luminex multiplex assays: IL-1α, IL-1β, IL-2, IL-3, IL-4, IL-5, IL-6, IL-7, CXCL8 (IL-8), IL-9, IL-10, IL-12p40, IL-12p70, IL-13, IL-15, IL-17, GMCSF, CXCL10 (IP-10), CXCL11 (I-TAC), IFNα2, IFNγ, TNFα, CCL5 (RANTES), Eotaxin, MCP-1, MIP-1α, and, EGF.
